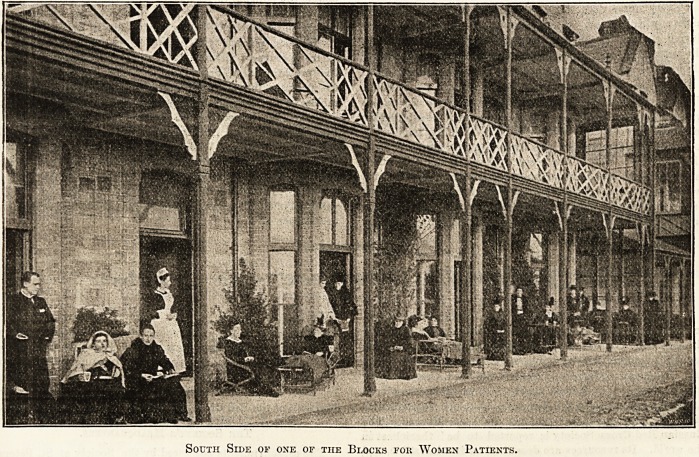# The Hospital. Nursing Section

**Published:** 1905-04-15

**Authors:** 


### The Hospital.

### Nursing Section.

Contributions for this Section of "The Hospital" should be addressed to the Editor, "The Hospital" Nursing Section, 28 & 29 Southampton Street, Strand, London, W.C.

No. 968.—Vol. XXXVIII. SATURDAY, APRIL 15, 1905.

### Notes on News from the Nursing World.

### THE ROYAL RED CROSS.

The King has conferred the order of the Royal Red Cross upon Mrs. K. Boyd. The distinction is bestowed by his Majesty in recognition of the services rendered by Mrs. Boyd, then Miss Driver, in tending the sick and wounded at the Volunteer Hospital at Intombi, near Ladysmith, during the South African War.

### THE PRINCE OF WALES'S NURSE.

During the recent indisposition of the Prince of Wales, he was nursed by Miss L. M. Goodwin. She was trained at the London Hospital and is now attached to the Nurses' Co-operation, 8 New Cavendish Street.

### THE DUCHESS OF CONNAUGHT AND THE NURSE.

In the course of her short stay in Rome the Duchess of Connaught found time to visit the Anglo-American Nursing Home. The visit took place on Sunday, April 2, and the Duchess was received by Miss Kirkpatrick, the directress, and some of the nursing staff. On the latter being presented to her, she noticed one of the nurses wearing the South African war medals. She seemed greatly interested and asked whether the mortality owing to gun-shot wounds or fever was the greater. Subsequently, the Duchess expressed herself as greatly pleased with the home and said that she had heard a great deal of the good work it had done for the Anglo-Americans visiting Rome. She was informed that a new wing for infectious diseases is in process of erection.

### THE "QUEEN'S NURSE" CERTIFICATE.

Miss Amy Hughes, who moved a vote of thanks to the officials of the Somerset County Nursing Association, at their annual meeting, must have been somewhat surprised at the speech of Dr. Edward Liddon, who seconded her proposal. Having stated that he would not be satisfied until every nurse in the employ of the Association had received a certificate of hospital training, Dr. Liddon said he thought that the "Queen's Nurse" certificate was merely ornamental. We entirely sympathise with Dr. Liddon in his desire that district nurses should enjoy the advantages of full hospital training; but Miss Hughes has probably already made clear to him the fact that the "Queen's Nurse" certificate is a very valuable and essential equipment for nurses engaged in district work. So far from the certificate given by Queen Victoria's Jubilee Institute being merely ornamental, it is an indisputable credential of the fitness of the holder to undertake the work of a district nurse.

### PRIVATE NURSING IN INDIA.

The article on private nursing in India, which appears in another column, will be read with interest by nurses who like the idea of undertaking work in our great dependency. Our contributor, writing with the authority of experience, gives some very excellent advice. But we are afraid that her article as it stands may convey the impression to thoughtless nurses that it is sufficient, if they have paid their passage and obtained their outfit, to have enough money in hand to maintain themselves for a month. This is not so. A nurse going out to India, or to any distant part of the world, to undertake private work, which may or may not be offered her, should have at least enough capital in her possession to last for four months. Before the end of that time she should be able to judge whether it is worth while remaining where she is, or whether it would be more prudent to remove to another part of the country.

### SLANDERING A QUEEN'S NURSE.

A case has just been heard at the Glamorganshire Assizes in which the plaintiff was Miss Margaret Jane Pugh, who has been a Queen's nurse for 10 years. The defendant is a contractor at a colliery at Six Bells, and he was charged with slander by Miss Pugh, the alleged slander being that he told two other men that she came to Six Bells with a false character, had been divulging medical secrets, and otherwise misconducting herself. The damages were laid at £500. The case for Miss Pugh, who has been engaged in nursing at the colliery for some time, was stated in detail; but the question of libel turned upon whether the words which constituted it were really spoken or not. The defendant was examined and cross-examined at length, and in the result a verdict was given for the plaintiff, damages £50. This verdict, of course, absolutely clears the character of the plaintiff from the imputations of the defendant, and will, we hope, serve as a warning to other persons in a similar position to refrain from lightly casting aspersions on nurses who have the right to expect every consideration from those by whom they are employed. The relations between workmen and district nurses are, as a rule, of a most satisfactory nature, and it is a matter for congratulation that an isolated attack on the reputation of one of the latter has been completely disposed of.

### MENTAL NURSING IN FRANCE.

The account given in another column by two English nurses of their experience in a large French hospital throws a lurid light upon the treatment of the insane on the other side of the Channel.

Judging by the description of the patients in the lunatic house, mental nursing as we understand it in this country is quite unknown, at least in one important French institution, and we are not surprised that when one of the English nurses entered the room containing the violent patients it reminded her of going into a cage. That in these days there should be men and women, even maniacs, chained to the wall, sitting with their hands tied behind them, or shut into a cold bath for seven hours in a foul-smelling corridor, proves how much there is to be done to raise the lot of the poor creatures above that of ill-cared-for beasts. We observe that the superintendent asked questions about English treatment. If French treatment of paupers mentally afflicted ordinarily resembles that which is in vogue at this particular lunatic house, it would be well if a number of French superintendents could pay a visit to Berry Wood or Claybury and realise for themselves what English treatment is like.

### RETIREMENT OF A LONDON MATRON.

Miss Mary Hull has resigned the post of matron of the Great Northern Central Hospital, which she has held since 1887. Her appointment, in fact, dates back from before the removal of the institution from Caledonian Road to its present site. Trained at St. Thomas's Hospital, she at once effected important improvements at the Great Northern Central. She first increased the period of training from one to two years, and subsequently, in 1893, to three years; and she has throughout discharged her duties with conspicuous energy and success. Applications from candidates for the vacant post are invited by the governors, and should be sent in before May 3rd to the Great Northern Central Hospital, Holloway Road, London, N. The salary is £100 a year.

### NEWPORT GUARDIANS AND PROBATIONERS.

At the last meeting of the Newport Guardians, the resolution increasing the number of lectures by the medical officer of the workhouse to the probationers by 10 to 15, per session was rescinded, and vacancies for ten probationers were filled. Prior to the four applicants for the appointments appearing before the Guardians, the chairman of the visiting committee expressed his conviction that they ought to know, "that the advertisement in response to which the applications were sent in was to some extent in the nature of a false pretence." He was afraid that the applicants thought that after they had gone through their course of three years they could take any position elsewhere. This, he added, was not the case, as they would not be qualified by their training for the office of superintendent nurse. In spite of this commendable frankness, there was no difficulty in filling up the vacancies. Newport being by far the most important town in Monmouthshire because of its docks and trade, the work at the Poor-law Infirmary is, of course, varied and interesting, though the training school does not take first-class rank.

### THE HOURS OF POOR-LAW NIGHT NURSES.

At the last meeting of the Woolwich Guardians Mr. Bentley called attention to the hours of the night nurses. He stated that they go on duty at 8 p.m., and remain until 7.30 a.m. This continues through the seven days of the week, and for three months at a spell. The nurses, he added, have only one night off per month to get any proper rest or sleep. It might, he knew, be contended that the nurses obtain rest and sleep in the day time, but he asked how it was possible for them to secure proper sleep with steam-whistles blowing, school bells ringing, and guns in the Arsenal firing? A short discussion followed, but the only remark which bore upon the question raised by Mr. Bentley was that of Canon Escreet, who pointed out that the Nurses' Home had been specially built in order that adequate sleeping accommodation might be provided for the nursing staff. The Home certainly fulfils the object for which it was erected, and from our own knowledge of the accommodation, we do not think that the night nurses at Plumstead Infirmary have any serious difficulty in obtaining their necessary rest during the day. As to the hours of duty, the fact that a proposal by Mr. Bentley for an inquiry on the part of the Infirmary Committee into the subject was adopted without dissent proves that the Guardians are willing to consider whether there is any substantial grievance which requires redress.

### THE LATE MISS S. M. ADAMS.

The funeral of Miss S. M. Adams, matron of Addenbrooke's Hospital, Cambridge, took place on Thursday afternoon last week at Wragby, Lincolnshire. Her remains were removed from the hospital on Wednesday morning, and a short service was conducted by the chaplain, the whole of the resident nursing, and domestic staff being present. The removal to the station was accomplished by the secretary-superintendent, Mr. Richard J. Coles, the assistant matron, and four of the sisters. Wreaths were sent by the committee of management, the nursing staff, the resident medical staff and the secretary-superintendent, and the domestic staff and porters, in token of their esteem. Though Miss Adams had been matron for only three months she had completely won the confidence of the committee, as well as the affection and esteem of the staff. The exceptionally tragic termination of a bright and useful life has been keenly felt at Addenbrooke's Hospital.

### MILITARY FUNERAL AT CANTERBURY.

A remarkable demonstration was witnessed at Canterbury last week on the occasion of the funeral of Miss Blanche Reed, a member of the Army Nursing Service Reserve. Miss Reed, who served with distinction during the South African War, gaining three medals, was transferred from Aldershot to Canterbury Military Hospital eighteen months ago. In the course of the winter she had charge of several cases of typhoid fever, and on March 18th she herself contracted the disease. From the first little hope was entertained of her recovery, and she died at the Nurses' Quarters of the Hospital on Monday week, at the early age of 33. Although attendance at the funeral by the soldiers was purely voluntary, the number who joined in the procession to the cemetery was the largest seen at any military funeral in the city for many years. Thousands of people lined the route, and the fine weather lent brilliancy to the animated and imposing spectacle. In addition to the relatives of the deceased, several members of the Army Nursing Service Reserve were among the mourners, and the large number of floral tributes included wreaths from Miss Sidney Browne, Matron-in-Chief of Queen Alexandra's Imperial Military Nursing Service, the matron and sisters of the Military Hospital, Dover, and the sisters of the Canterbury Military Hospital.

### NURSING CONSUMPTIVES AT VENTNOR.

The details of the duties of the staff at the Royal National Hospital for Consumption, at Ventnor, which are given by the matron to our commissioner, indicate that the work is very suitable for educated young women who desire to gain some knowledge of special nursing before they enter a general training school. The hours are about the same as in other institutions, but the nursing is not so arduous. The surroundings are exceptionally pleasant, and the fresh air and exercise should do much, by building up their health, to enable them later to stand the strain of a great hospital or infirmary. When the nurses' home, which we are assured the committee are anxious to provide as soon as funds will permit, is added to the splendid pile of buildings along the seashore at Ventnor, the conditions will leave nothing to be desired.

### THE NURSES OF THE EAST LONDON HOSPITAL

In the building containing the new whooping-cough wards at the East London Hospital, opened on Saturday by Princess Louise of Schleswig-Holstein, provision has been made for a sister-in-charge and six nurses to be in attendence on the 12 patients. The rooms provided for the staff are both light and well ventilated, each bedroom having a fanlight over the door, while there is a ventilator also at the end of the corridors. The dining-room, on the entrance floor, is simply and artistically furnished in fumed oak; and there is a pantry adjoining, for making tea, etc. A prettily-furnished bed-sitting-room has been allocated to the sister-in-charge, and this, like the nurses' bedrooms, is also fitted up with fumed oak tables, chairs, and hanging-press. The little leather-topped writing-tables, one in each of the bedrooms, will prove a boon to the occupants. The floors, on which rugs and mats are spread, are of teak, and the walls, like the entire building, are painted pale cream. The electric light has been installed, and provision for escape in case of fire is effected by an outside iron staircase. The tastefully-arranged pots of daffodils and other spring flowers added greatly to the attractiveness of the new home on Saturday afternoon.

### DISTRICT NURSING IN HOLLAND.

A melancholy interest attaches to the account which we publish to-day of a chat with a Dutch district nurse. Since our correspondent visited Sister Salm she has passed away, having succumbed, after much suffering, to rapid phthisis. She was only 41, and was greatly esteemed by all classes in Haarlem. The details of her busy and useful life will be read with the attention they deserve. They correspond, in the main, to those of the ordinary district nurse in this country, and it will be observed that Sister Salm was fully trained and qualified as a monthly nurse. Her daily visits to the school children of the church to which she was attached was an unusual feature, and perhaps not the least important of her manifold obligations was her constant attendance upon the old people in the almshouses.

### NURSING HOMES AND NURSING INTERESTS.

If it were not that almost every week brings us letters from nurses asking for advice and sympathy, we should hesitate to reiterate so frequently a warning against accepting employment in private institutions without due inquiry. The title "Co-operation," which has been used by several institutions since the Nurses' Co-operation, 8 New Cavendish Street, has proved so successful, has been the means of misleading nurses. The title guarantees very little, and its adoption by an institution is no reason why a judicious inquiry should be withheld.

### OPENING OF A CENTRAL HOME AT ADELAIDE.

The South Australian branch of the Royal British Nurses' Association has entered into possession of its central home in Adelaide. The home has been established for members who are engaged in private nursing, in order that they may be able to live there at the cost of a moderate fee for board and residence, and also for the benefit of those who are sick and are without homes of their own. The latter can be received free of charge until they are well enough to resume their nursing duties. Any nurse visiting Adelaide can also use the home as a boarding-house at a charge of 15s. a week. The home, which was formally opened by Sir George Le Hunte, the governor, and Lady Le Hunte, is exceedingly well situated and is surrounded by spacious grounds. The property belongs to the Government, and was formerly the residence of one of its officials. The accommodation is admirable, and there is an excellent tennis court. Miss Knowles, the matron, has with her in residence eight members of the Association, and the start thus made by the Royal British Nurses' Association in Adelaide is sufficiently favourable to justify hopes of a permanent success.

### NURSES WHO DON'T KNOW THEIR OWN MIND.

Newton Abbot guardians have resolved upon taking unusual action in regard to the recent appointment of a nurse. On several occasions they had the experience of interviewing and appointing nurses who afterwards wrote declining the situation, and they think it is time such capricious behaviour should come to an end. A nurse from Southampton who was appointed to a vacancy on the staff, a week later wrote that she desired to remain where she was, and offered to refund the travelling expenses paid to her. It was suggested that the guardians should claim the cost of advertising again, but eventually they decided to require her to pay a month's salary in lieu of notice, which comes to about the same amount. On the other hand, the Exeter guardians, after actually sending out advertisements, have received a letter from a nurse asking to be allowed to withdraw the resignation which she had previously tendered.

### The Nursing Outlook.

"From magnanimity, all fear above;
From nobler recompense, above applause,
Which owes to man's short outlook all its charm."

### THE AFFILIATION OF TRAINING SCHOOLS.

The first week in May should mark a new departure in advance for the education and training of nurses in the United States. The annual conference of the Society of Superintendents of Training Schools and Associated Alumnæ will be made the occasion for the consideration of a number of papers of great interest to all who are engaged in nursing, and also to the teachers and managers of nurse-training schools. Mrs. Hunter Robb, who was for several years the able superintendent of the Johns Hopkins Hospital, Baltimore, has promised a paper on the affiliation of schools for educational purposes. In this paper she proposes to show how such affiliation can best be attained, how existing standards and varying requirements can be adjusted, and to what extent and why affiliation is called for in the best interests of higher nursing education, and the most representative nurse-training schools. We shall look forward to the report of the proceedings at Washington with great interest, for Mrs. Hunter Robb's paper may mark a new departure leading to important results.

In England as in America it is beginning to be more and more recognised that some step should be taken by affiliation to utilise the material offered by special institutions for the completion of special courses by probationer nurses preparing for their final examination and certification. At the Seamen's Hospital, Greenwich, Queen Alexandra's Imperial Nursing Service objected to the certificates issued by the "Dreadnought," on the ground, that the nurses had no adequate training in the diseases of women. In order to overcome this objection the "Dreadnought" Committee have arranged with some of the women's hospitals to take their probationers for one year and to train them in this special branch of their work. Again the higher education of nurses entails adequate provision for theoretical and practical instruction in a number of subjects, which have not heretofore been entirely covered by the majority of nurse-training schools. Medical and surgical nursing have formed the main subjects, but, as higher education has developed, a demand has arisen for special facilities for instruction in obstetric, fever, children's, eye, light and electrical cases, as well as in district and private nursing. The special hospitals in the United States and in this country have sometimes experienced a difficulty in securing the services of sufficient nurses to fulfil their requirements. It would therefore appear, that, from the standpoint of higher education, the affiliation of nurse-training schools with each other and with certain special hospitals is much to be desired.

The first step to be taken is undoubtedly to secure the collection and arrangement of complete information as to the existing conditions and requirements of nurse-training schools, special hospitals, and the nurses themselves. This might be usefully undertaken by the Central Hospital Council, which has already done some useful work in regard to the systematic training of nurses, through a committee, which might well be re-appointed to deal with this important matter. No intelligent manager of a nurse-training school in this country can doubt, for one moment, that the present isolation is harmful to the interests of each and all the nurse-training schools. There are many reasons, apart from the higher education of nurses, which render it desirable that some steps should be promptly taken with a view to affiliation. The Central Hospital Council which has yet to win its spurs by the accomplishment of more practical work, might therefore welcome the suggestion we have ventured to make, for, by its adoption, they might reap no little credit by helping each and all the nurse-training schools to help themselves in a practical way.

The Central Hospital Council's Committee has pointed out the desirability of levelling up the existing methods of instruction and examination, so as to make the certificates issued, by each school, represent something like an average unit of efficiency. There is no reason why one school should differ materially from another in its methods of training, and in the conditions it imposes upon probationers before they can secure a certificate. The more the authorities of the nurse-training schools become accustomed to co-operation, the greater will they find the advantages which are likely to accrue to each and all, from the adoption of some such system. There is, at the present time, a consensus of opinion in favour of some general step in the directions indicated, and we believe that the proposals of Mrs. Hunter Robb, resting, as they no doubt will do, upon accurate information clearly arranged and recorded, must tend to directly help forward the movement in the United States, and indirectly to promote the same end on this side of the Atlantic. What is most needed, at the present time, is the provision of the maximum facilities for all candidates for the higher nursing certificates, to enable them to readily conform to the requirements of the nurse-training schools. No training school, in fact, can reap anything like the same advantages from isolation that would accrue to it from affiliation. As a mere matter of business, therefore, if the Central Hospital Council were to take the initiative, affiliation might be an accomplished fact, in this country, before the present year is out.

### The Nursing of Sick Children.

By James Burnet, M.A., M.B., M.R.C.P.Edin., Registrar, Royal Hospital for Sick Children; Clinical Tutor, Extramural Wards, Royal Infirmary; and Physician to the Marshall Street Dispensary, Edinburgh.

### IV.—ON THE MANAGEMENT OF RESPIRATORY CASES.

By respiratory cases, we mean those who are suffering from such common affections as laryngitis (inflammation of the larynx), bronchitis (inflammation of the bronchial tubes), or pneumonia (inflammation of one or both lungs). Much depends on the nursing of such children, for if they are badly attended to they not uncommonly succumb, whereas if carefully nursed they can, as a rule, be brought through what may prove to be a very serious illness.

The medical attendant always forms a favourable impression of the nurse who knows exactly *how to prepare the patient for examination.* In mild cases the child should be made to sit up in bed, his back being supported by a pillow pressed against it by the nurse's hand, and a shawl kept over his chest while the physician examines the back. If the patient is too ill to sit up, he should be gently turned over on to his side during the examination. In the case of infants, the nurse should keep the patient on her knee with a blanket or shawl wrapped loosely round him. As the examination in such cases is generally conducted before a fire, the nurse should see that the patient's body intervenes between the physician and the fireplace, otherwise the former will have his face scorched during the process. Nurses are often very thoughtless in regard to this important detail, and so we mention it particularly in order to put our readers on their guard.

*Position of the Patient.*—No attempt should ever be made to make the patient assume a position in bed which he does not appear to desire, otherwise discomfort and consequent fretfulness will result. If the child appears to breathe more easily while sitting up in bed he should be allowed to do so, but he must be packed closely round with blankets and have his back supported by pillows. A thick shawl or blanket should also be thrown over his shoulders. In the case of feeble patients, especially infants, the nurse should see that the patient's position is changed from time to time. This is a most important matter, as congestion of the lungs and collapse of portions of these organs may supervene if the child is kept too long in the one position. Infants should be lifted from their cot and kept on the nurse's knee before the fire for a certain period each day.

There are four important matters to be attended to in the nursing of respiratory cases, and these we shall now briefly consider. There is first the *cough.* When the larynx is principally involved the cough is peculiar and assumes the well-known croupy or hoarse character. In bronchitis it is harsher and may at first be dry and short, but soon it becomes looser, and is apt to come on in paroxysms. It must be carefully distinguished from the cough which is met with during the course of whooping-cough. In the latter disease we have it associated with vomiting, and it is always much more paroxysmal and prolonged than is the case in ordinary bronchitis. The cough of pneumonia is typically short, dry, and restrained, the child evidently trying to keep from coughing on account of the pain which the act occasions. When the cough is very severe a warm drink often gives relief, and should it prove very troublesome the patient must be kept propped up and supported by the nurse until the attack is over. A few drops of glycerine in water is often soothing. It should also be remembered that draughts of cold air very often induce a fit of coughing. This may be brought on by the nurse coming from the outer air straight to the patient's bedside, or by simply opening the door of the sick-room suddenly.

The cough is usually accompanied by what is technically termed the *expectoration.* In babies and young children, however, what is brought up from the respiratory passages is never really expectorated from the mouth, but is immediately swallowed. Sometimes the material brought up is very thick and sticky in character. This is especially so in cases of pneumonia, and the nurse may have to remove it from the child's mouth with her finger covered over by the corner of a handkerchief. In the earlier stages of all respiratory diseases the expectoration is always scanty, but as the case progresses it becomes more copious. It may contain blood, and this spitting of blood is technically termed hæmoptysis. This phenomenon, however, is comparatively rare in children, and is almost unknown during the period of infancy.

The third symptom of which note must be made is *dyspnœa.* This simply means rapid and difficult breathing. If the child has actually to sit up in bed in order to obtain breath, the condition is then termed orthopnœa. When the breathing is greatly embarrassed the lips may become purple in colour, and the condition known as cyanosis is then present. Patients in this condition require very careful management, and if immediate relief is not obtained they very often die quite suddenly.

*Pain* is the last of the four special symptoms to which we wish to refer. The nurse should, if possible, note when this occurs, whether it is only present during coughing, or seems to be constanty felt. Children very often are unable to say where the pain is, and not uncommonly point to the abdomen when the pain is actually in the chest. In cases of pneumonia, however, abdominal pain is often present at the commencement of the attack, a fact which is apt to lead the inexperienced to the conclusion that the disease is not one of the respiratory organs at all.

The nurse should always report to the medical attendant any variation in these symptoms which she may observe from time to time, such as an aggravation of the pain or of the cough, or any increase in the dyspnœa as evidenced by indrawing of the spaces between the ribs and of the parts at the root of the neck. She should also observe whether or not the nostrils move during respiration, as if they do a considerable amount of respiratory difficulty may be assumed to be present. The amount of nourishment taken should be carefully noted, and the periods of sleep should be specially reported.

The bowels must be carefully attended to, and in children who are very feeble or even semi-comatose, the evacuation of the bladder should be watched for. If no urine is passed for twelve hours the fact should at once be reported to the physician. Constipation is apt to produce flatulence, which very frequently leads to considerable embarrassment of the respiration. The greatest possible care must be exercised in the feeding of such patients. Should a fit of coughing come on no attempt should be made to give any food. Even when the breathing is difficult careless feeding may result in the child's being choked. In the case of infants the nurse should see that the food is not given too quickly. In many cases spoon feeding must be resorted to, and even then there must be no undue haste displayed in feeding the child. If the food is vomited shortly after it has been given this generally means that too much has been fed to the child or that it has been fed too rapidly. Fits of coughing, however, often lead to vomiting, and in such cases the nurse should endeavour if possible to avoid giving the patient much nourishment until the paroxysm has completely passed off.

The temperature of the sick-room should be maintained at an equable height of about 70° Fahr. throughout the course of the illness. This temperature must be kept up during the night as well as during the day. Care should be taken to see that the child does not throw off the bedclothes while asleep, as he may thereby contract a chill which may lead to an aggravation of his illness, or even to a recrudescence of the disease during convalescence.

### The Nurses' Clinic.

### MAMMARY ABSCESS. BY A SUPERINTENDENT OF DISTRICT NURSES.

District nursing does not as a rule include ordinary maternity work, but cases are always accepted if such complications as white-leg or mammary abscess have arisen.

In the case that I am about to describe we were not sent for until the breast had been lanced by the doctor to allow the discharge to escape freely. This most necessary operation is sometimes opposed by the patient's friends, with the result that recovery is retarded and health permanently weakened. The nurse must assist the doctor by bringing all her influence to gain their consent, assuring them that lancing will mitigate suffering and hasten recovery, and describing the disastrous results that will ensue if they persist in their refusal.

There are doctors (and nurses!) who when they first start work among the poor are filled with the desire to give all the patients exactly the same things that they have in the best-provided hospitals. About a year ago I met a doctor at a lingering, hopeless cancer case. He was supplying the woman at his own expense with iodoform gauze, wool, tabloids, etc. He was young, and I said to him warningly, "You will hardly be able to go on doing this." With the enthusiasm of a beginner he warmly dissented, "Ah," I said, "the force of circumstances will bring you, as it has brought us, to clean rags and boracic lotion." Time passed on. We sent to his house, as directed, for the dressings, but ever smaller and smaller portions were given, and at last came a day when the husband heaped insults on him for giving so little! The next time I saw the doctor he said, "Do you know of any home into which we could get the poor woman?" Happily I did know of one, a home for the dying, and there the sufferer ended her days in peace. The moral is: Do not raise expectations which you neither can nor ought to fulfil. "Justice is a higher quality than generosity." We should no more be eager to take other people's rightful responsibilities upon ourselves than to shift our own on to other people. Often when I go to a surgical case, the friends say to me, "I suppose you have brought the dressings with you, nurse?" "No," I reply, "I have brought you my ten fingers and all the skill that is in them. I can tell you where to buy the things," I add, in the tones of a person making a great concession and parting with a professional secret, "and how much they cost." They gaze at me as I sit bland and inactive; finally they laugh, and go out to fetch the dressings.

The treatment ordered by the doctor in this case was, the wound to be well syringed with boracic lotion, a boracic poultice to be applied covered by wool and a mammary abscess binder. We told the girl who went to fetch the necessary articles to buy a pound of boracic powder at 8d. and a packet of wool at 10d., a yard of flannelette to cut into two triangular bandages, and half a yard to cut into rounds with a diameter of about 11 inches. The bandages should be lightly hemmed all round, and the circular pieces must be pinked at the edge, lined with wool, and have two tapes sewn on at opposite sides. A piece of jaconet with a diameter of 10 inches should be tacked on to the flannel. Tapes must be sewn on the triangular bandages at a distance of three inches from either side of the centre of the base, and tapes may also be needed at the two lower corners.

A basin should now be put into the oven, covered by an old plate, and a strong piece of rag should be kept to be used every day as a wringer.

The table must then be arranged in strict order as for a surgical dressing. If nurses would only adopt the habit of doing these things systematically they would do them more rapidly and satisfactorily, and have their minds more at leisure to meet the irregular but most important claims continually made in district nursing. Let me once more remind nurses never to lay their instruments down on table or bed, but to put them back in the lotion bowl however often it must be done.

Having dressed the wound and placed a guard over it, prepare the fomentation. The fomentation should, if possible be of four-fold lint, but if strict economy is necessary all but the fold next the skin can be of white flannelette or clean soft rag. A hole must be cut in the centre for the nipple. Make the wringer dry and hot and put it in the basin with the lint put in one teaspoonful of boracic powder and then add the boiling water. Wring the fomentation tightly, pour the water away, and take the fomentation to the bedside in the basin covered by the plate. Apply the lint, cover it with the wool and mackintosh that has been tacked together, tie one tape under the axilla of the affected side and one round the neck. Put on the triangular bandage, tying the tapes that have been sewn on the base round the waist. Place the apex over the shoulder of the affected side, pass one end of the base round the neck and one under the axilla of the affected side, tie these two together; pass the apex under the knot, draw it up gently until the bosom is well supported, then fasten with a safety pin and place a piece of wool under the knot. All this sounds lengthy and troublesome, but in reality can be carried out quicker than any makeshift lack system.

### A Novel Mode of Nursing the Sick in the Far West.

BY AN ENGLISH NURSE.

We were ready to start for two months' sojourn at the coast at a village called Morro. Our party consisted of five, our patient, a young Englishman suffering from nervous prostration and on the verge of consumption, brought on by his own folly—a good-hearted young fellow, who bore out Tennyson's words, "Lord of himself, that heritage of woe;"—his sister, a sensible, jolly little soul, ever ready to help; next, our Jehu, the personification of "Uncle Josh," though not with his cheerful disposition and aptitude at enjoying a joke on himself; then our male nurse, a great favourite always with nurses and patients, and just the one to help with the patient; lastly, myself, a trained nurse with considerable experience in camping life, both with sick and sound. Our travelling conveyance was a large camping waggon with a canvas top to keep out rain or sun. Our male nurse drove the light waggon at the back with the mattresses. We were all attired in more or less dust-coloured garments of the coolest description, for we had to be prepared for clouds of dust and a tropical sun. We had five days of pleasurable discomfort ahead of us, and we all tried to take it in a "Mark Tapley"-like spirit. We really did look a queer lot, togged out as we were in our oldest clothes. We did not load up in front of the sanatorium; not, however, for the reason given by John Gilpin, "lest folks should say that we were proud," but because we did not want to attract the public gaze. Our patient sat on the front seat with "Uncle Josh," and his sister and I occupied the back seat, with our feet, where we could find room for them, between pots, kettles, hold-alls, etc. Although only 4 a.m. when we started it was quite hot, and we settled ourselves as comfortably as we could, for we had to go 15 miles before we rested. The first few miles were through familiar scenery, peach and apricot orchards on each side of the road, then long stretches of fields of alkali land, only relieved by the tall sunflowers springing up in patches and salt grass.

### The First Halt.

The monotony was interrupted by sundry stops, occasioned by our male nurse shouting from the rear to the effect that the kettle had got loose from its moorings and was reposing in the dust 50 feet away; that the hat of our patient's sister which she had pinned on the canvas, had fallen off, and the grey horse at the back was unintentionally holding it down with its hoof, unconsciously rendering it more fashionably flat than before; or that the barley was leaking out of the sack by reason of the jolting of the axe's sharpened edge against it. At last, coming to a shady spot near an irrigating ditch full of running water, we resolved to make a halt, unhitched the horses, and fed and watered them. The male nurse and our patient collected sticks and built a camp fire, the ladies turning for the nonce into cook and housemaid, and soon had a presentable dinner ready. It was rather smoky, and consisted only of fried bacon, new bread and coffee, melons and peaches. But it was the daintiest meal we were destined to enjoy for five days. When we had finished we washed up our dishes in the stream, then packed up, and, the horses being refreshed, we started off, and only halted once until we had made about 20 miles more at Hanford, the last town we were to pass.

### Our Last Glimpse of Civilisation.

Every mile brought us into more uninhabited country, and the male nurse, by aid of his gun, was able to provide us with a supper of wild doves and cotton-tails, a small kind of wild rabbit. Towards evening we arrived at King's River, a very picturesque spot, and decided to camp for the night. This was our last glimpse of civilisation for two days, until we got through the desert. The place consisted of a store containing canned goods, and a saloon or public-house for the convenience of campers, and a cottage and barn. Whilst the patient and his sister and "Uncle Josh" unloaded the bedding, the male nurse and I built the camp fire, and he being a good cook, prepared his game and cooked it, whilst I laid the cloth. "Uncle Josh" disappeared soon after, and returned smelling strongly of the "cup that cheers," but also inebriates, and he got quite rusty when I put down my foot on his wishing to "treat" the "sick gentleman," who behaved very well over it, and listened to the advice to have a good cup of black coffee instead. When the friendly darkness threw her mantle over us, the patient's sister and I slipped away to change our camp dresses for warm dressing-gowns, and to get a cooling wash in the rushing river, and, returning, found the others already wrapped in peaceful

The Start.

slumber not far off, "Uncle Josh" rivalling the coyote howls in the distance with his unmusical bass snore. We were soon asleep, until in about what seemed 10 minutes I was awakened by "Uncle Josh" ejaculating, "It's just three by my timepiece." So we made an enjoyable but somewhat gritty repast, and started to cross the desert just as dawn was breaking, after carefully filling the demijohns with water.

### In the Desert.

For the first few miles the monotony and lonesomeness of the desert did not pall, but when out of sight of all but sky and sand and sage brush, we began to grow quiet, and our patient, who had up till then given us no trouble, commenced to cough and to regret that he had been led into making the trip. It required all our tact to make things go at all smoothly, and I began to fear that we were going to have the same trouble we experienced on our last camping-out trip; but, as Kipling says, "that is another story." The heat waxed more and more intense, and occasionally we saw skeletons of cattle, sheep, or horses which had strayed and fallen by the way. Sand, sand, sand, under a pitiless sun, with nothing to vary the monotony. The horses never went off a walk that day or the next, and at times we had to stop to sponge out their mouths. About noon we came to some artesian wells, a spot in the desert where the water gushes up and is pure and refreshing. By the side of the well were two or three tumble-down wooden huts, and an old barn full of hay, the only inhabitant being an old fellow with a wooden leg, who eked out a scanty living by selling hay and bad whisky to passers by. "Uncle Josh," of course, patronised him, and nearly got bitten by one of the two dogs belonging to the place. There being no wood wherewith to make a fire, we contented ourselves with cold food and lemonade, and even these we could hardly take by reason of the swarms of flies. We rested until three and then left, plodding on in the blinding heat hour after hour, occasionally drinking out of the demijohn; but the water, so cool when we filled it, was now lukewarm. Towards night we arrived at a sheep corral, and the shepherds, a rough-looking lot of men, let us put down our mattresses in a corral they were not using, and, fater a makeshift meal, we lay down unwashed and weary.

In the Desert.

The only disturbance we had that night was when a hungry goat attacked my hair under the impression that it was hay. All the next day the same experiences attended our journey, only we came across some sand hills, which varied the monotony, and to relieve the horses we got out and walked.

### Vicissitudes of the Journey.

"Uncle Josh" developed a particularly lugubrious turn of mind, and, after a long silence, in which he spat and chewed alternately, he growled out, "Waal, I'll be darned ef we hain't lost our way. I reckon we won't be the first who's died of thirst if we don't git ter water." Soon after one of the team of horses went lame, and we had to halt for two hours. We made the rest of the lukewarm water into lemonade, saving about two quarts to wash the horses' mouths. Towards night a breeze sprang up, and by mid-night we got to Cottonwood Canyon, an old Indian camping-ground, where there was one spring of lovely cold water and another of sulphur. The patient looked very ill, so the male nurse and I did our best to make him comfortable with a cup of tea and a rub down, the best we could do under the circumstances, and we could see he tried to bear up. The shepherds were all Portuguese and Mexicans, and looked as if they would cut anyone's throat with pleasure. "Uncle Josh" refused to sleep out of the waggon for fear "that durned Mexican trash should knife his innards," and had such a fit of ill temper on that we almost felt inclined to do it ourselves. He would not help in cooking, and yet ate the chief part of the watercress we had taken great pains to gather in order to tempt our patient.

### Adventure with a Scorpion.

Next morning we arose at 4 A.M., feeling better and more cheerful as each step took us into fresh and beautiful scenery. The two men procured us a good dinner and supper with their guns. The water all along the road was now plentiful, there being mountain springs every few miles, and having all the fire-wood we wanted at our command we made a rousing bonfire and slept on our mattresses around it. This fire is necessary, for though it is seldom that wild beasts cross a travelled road, there are plenty of bears, pumas, lynxes, wild cats, and rattlesnakes in the mountains, and attacks from such animals are not unknown. In the morning, it being cool, I was about to put on my coat, which I had used as a pillow, when the patient jerked it off, and, turning, I beheld an angry scorpion walking out of my sleeve. He had the laugh then, and declared that it was I that had nervous prostration. Next night we slept at San Luis O'Bispo, but the camping-ground was infested with fleas, and we were all glad that we had only 18 miles more to journey before reaching our destination. As we went over the last hill, a most glorious view of the Pacific Ocean met our gaze. A huge towering rock stands about 300 yards out in the ocean, from which the village takes its name, and to the south, surrounded by the hills, was a beautiful bay, as blue just then as the Bay of Naples.

### The Cure of the Patient.

Early though it was in the season, already white tents were visible amongst the wooden camping huts. We first decided on a furnished hut, of which the owner gave an encouraging description, but on arriving at it and finding the furniture consisted of a cooking stove that on lighting smoked so that we had to sit outside, a coffee pot with a hole in it, inhabited by a deadly black spider, and three rusty tin plates and a dirty feeding bottle, three wooden boxes, and a rickety table, and a "spring mattress," very dirty, with the spiral springs peeping out in derision all over it, we decided to take an empty house and furnish it ourselves. Our first meal in a house was simply delicious after our open-air picnics.

I have only to add that at the end of ten weeks we returned home, our patient having added 22 lbs. to his weight, being the picture of health and manliness, the threatened consumption a thing of the past, and the craving for drugs and drink entirely eliminated. One small ache, it is true, was left which had not been there before; but I think a little Spanish girl, whom I noticed just before the road turned with a handkerchief to her eyes, could tell more about that than his nurse.

### To Nurses.

We invite contributions from any of our readers, and shall be glad to pay for "Notes on News from the Nursing World," or for articles describing nursing experiences at home or abroad dealing with any nursing question from an original point of view according to length. The minimum payment is 5s. Contributions on topical subjects are specially welcome. Notices of appointments, letters, entertainments, presentations, and deaths are not paid for, but we are always glad to receive them. All rejected manuscripts are returned in due course, and all payments for manuscripts used are made as early as possible after the beginning of each quarter.

### The Work of the Deaconess-Nurses at Düsseldorf.

BY AN OCCASIONAL CORRESPONDENT.

Through the courtesy of the English chaplain at Düsseldorf I had the opportunity of visiting the "Evangelisches Krankenhaus" of that flourishing up-to-date German town. The matron asked me to wait while she summoned the only English sister resident in the hospital. After a long pause a young sprightly sister appeared, attired in the dress of a Kaiserswerth deaconess; and it was droll to see an English —or rather, as it proved, an American—face framed in the sober, old-fashioned deaconess cap. But this was merely the first of the surprises I experienced that afternoon. I learnt that the entire nursing of the hospital was in the hands of the Protestant deaconess-nurses of Kaiserswerth, and my admiration and amazement grew greater as their self-denying work was rapidly unfolded by my lively young guide, who assumed the *rôle* of cicerone with evident relish and enjoyment.

### The Wards.

The building consists of three blocks, the administrative portion being in the centre. The hospital provides accommodation for 200 patients in the main building, in addition to about 20 beds in separate departments for the treatment of typhoid and diphtheria cases. The wards all open out of long corridors, and, having only a large window at one end, are not as well ventilated as if there were a cross-current of air through them. There are usually 12 beds in each ward. The bedsteads are of iron, but with a great deal more mattress than we think necessary in England. The floors are polished, and a little table stands beside each bed. The usual black slate hangs at the head of the bed, with the name and religion of the patient writ large upon it. Temperature charts are conspicuous by their absence; only in very bad cases is the temperature recorded daily.

### Paying Patients.

Everywhere upon the Continent one sees a great lack of colour in hospital wards, a long monotony of blank, staring whiteness, which strikes a stranger's eye somewhat unpleasantly, and one thinks with relief of our own cheerful red screens and scarlet bed-jackets. Attached to the women's wards are private wards for first and second class patients. Of the former there are two classes, who pay respectively 10s. and 8s. a day. The second-class patients pay 3s. a day, and must not object to occupy a double-bedded ward. The third-class pay about 1s. 6d. a day. The first-class wards are quite elegant, with brass bedsteads, good suites of furniture, chairs, and couches upholstered in crimson and other etceteras of doubtful advantage to a sick person according to English ideas. There is only one ward-maid to each women's division, and no probationers in our sense of the word.

### The Sisters.

Each nurse has her separate department, and does practically every duty connected with it. My guide told me that she had a ward of 12 beds and four private wards to manage quite alone; even cleaning the floors is done by the nurse. There are 32 sisters in the hospital, but they are by no means all nursing sisters. One superintends the laundry, another the kitchen, etc., etc. I fear the word superintend hardly conveys the right impression—it is a case of turning up sleeves and actually doing a large share of the work. The kitchen department is a very heavy responsibility, as paying patients require much fadding, and frequently refuse what is sent to them, and then sister rushes to the kitchen and implores the presiding genius to find something else for her patient, and the long-suffering kitchen sister has to produce something appetising at the shortest possible notice. All the cooking is done in the basement. The kitchen and scullery have tiled floors and partially blue and white tiled walls; a large stove such as is found in all continental hospitals, with flues beneath the flooring, is used for frying and baking purposes. There are three kitchen-maids and three scullery-maids—the latter prepare vegetables. This latter operation is not so simple a matter as with us, as the Germans like everything seasoned more or less; and I was told that it is not uncommon to give patients considerable latitude in the way of food—in short such unsuitable comestibles as would make an English nurse's hair stand on end if she saw it. But then digestions differ according to nationality. It must be so, otherwise serious results would follow such proceedings.

### The Question of Diet.

The patients' meals are served as follows:—At 6.15 a.m., coffee, bread and butter; at 9 a.m., bouillon and sandwiches made of sausages, or ham with bread and butter; at 12 noon, soup, meat, vegetables, rice and fruits; at 3.30 p.m., coffee and rolls and butter; at 6.30 p.m., milk and rice, some kind of cakes or scones fried in fat, or macaroni and prunes. The first class of course have additions to their diet, and it is served with more nicety and daintiness than the second and third class meals. The diet of fever patients is not quite so liberal as I have quoted, but it struck me as being throughout on a very liberal scale.

### Hours of Working.

The sisters rise at 5.30 a.m., prayers are at six, and they go on duty at 6.15. There is positively no off-duty time; each nurse goes to bed when she has finished her work in the wards. Think of such a life of self-denial, my fellow-nurses, and be thankful for more merciful regulations where our lot is cast. If a bad case requires a special nurse, the nurses divide the extra duty between them; one sits up till 2 a.m., and rises again at 6 a.m. as usual. This my informant told me did not occur more than once a week! The nurses' meals are served on an open-air balcony when the weather is suitable.

### A Church Bedroom.

A disused chapel has been utilised for sisters' and nurses' bedrooms. The middle aisle is now the corridor, with rooms on either side. Each room is occupied by two sisters. They are comfortably furnished. The one at the extreme end rejoices in three stained windows, and the occupants must have the feeling of sleeping in a church. It has a very quaint appearance, this queerly-shaped room. I had heard of a church parlour, but not of a church bedroom; no doubt, however, the sisters sleep well in "an odour of sanctity."

### One Night Nurse.

There is only one night nurse to the entire hospital, except in cases of severe illness, or operation, or dying cases. Then a special nurse is put on. This seems extraordinary, but it is not so dreadful as it seems, as the night nurse does no washing of patients or making of beds, nor any preparation of breakfast for the patients. She merely walks round the building once in every hour, and attends to every bell that rings during the night. The nurses in charge of the typhoid and diphtheria wards sleep near their patients and take entire charge of them. One nurse often has charge of several tracheotomy cases at the same time, and apparently lives without going out or seeing her fellow-creatures except through the windows of her bungalow.

### Male Nurses.

The male patients are attended to by male nurses, who are trained specially and very well paid. The sisters merely have the general superintendence of the male wards, take temperatures, and give medicines, etc. No objection is made by the authorities if a lady wishes to enter the hospital as a nurse-probationer, but if so she will only learn nursing and may decline to help with the ward work altogether, so that one can understand that the deaconess-nurse by no means welcomes a lady probationer.

### The Dispensary and Linen-rooms.

The dispensary is in charge of a qualified sister, who has a great deal of work to do, without any assistance except a maid to clean the floor. She looked tired and worn, sadly needing a holiday. As I gazed at the bottles on the shelves I was glad that the familiar Latin names could not be made over into German; the smells and odours were familiar too. The matron herself showed me the linen-room, where piles of snowy sheets and serviettes reposed on spotless shelves; every article was folded with military precision, and I admired it all to the visible satisfaction of the good Sister.

### The Operation-room.

But the most interesting sight of all was the operation-room; this is well-nigh perfect in every detail. A large square room, very well lighted and ventilated, with tiled floors and walls, white ceiling, glass cupboards full of shining steel instruments, glass tables and vessels, and exceedingly light and up-to-date operation table, stands for lotion bottles, liberal supply of lavatory basins, all of shining whiteness, tin trays for bandages and dressings, and long white pinafores with short sleeves for the surgeon's use when operating. There is a sister set apart for this department, and I observed that a maid was helping to clean up after an operation. The electric light is fixed in the ceiling exactly over the operating table. All patients are anæsthetised in an adjoining room, so that they never see the preparations made for their benefit. A rather smaller room is reserved specially for septic operations and for the dressing of major wounds. Minor dressings are done by the nurses in the wards. Ample arrangements are provided for sterilising instruments and dressings. On the ground floor is a Roentgen-ray apparatus and all the paraphernalia of the Finsen light cure.

### General Features.

Quite recently balconies have been affixed to the front of the building, so that patients can be wheeled into the fresh air, and enjoy a sight of the garden which is gay with flower-beds of brilliant colours. There is also a charming garden at the back of the building for the benefit of convalescents. From the report of the "Evangelisches Krankenhaus" I learnt that a peculiar arrangement exists for the benefit of domestic servants. A yearly payment of 12s. insures the admission and medical treatment of a man or woman servant for a fixed period, otherwise the laws of Germany compel the master of the house to pay the doctor's bill as he may not send away a servant ill. Gynæcological work is a great feature in the surgical department, and is under the care of an eminent surgeon. Very good results are obtained from the large number of yearly operations. A special operation-room is set apart for ophthalmic work, where, during the year, no fewer than 133 operations were performed by the ophthalmic surgeon. Cases of cholera, small-pox, epilepsy, syphilis, and mental cases are not admitted. A certain number of beds are reserved for gratuitous treatment, and in spite of the fact that the majority of the patients pay something towards their maintenance, the expenses far exceed the income, funds, as in England, being urgently needed.

### Tending Sick Natives in Algeria.

### THE PRESENT POSITION.

The civilising and humanitarian policy of the French Government towards the native races of its great colony is in no way better exemplified than in the establishment and rapid multiplication of hospitals specially adapted to their requirements.

### The Arab Hospitals.

There are, of course, several large mixed hospitals in the different towns, but it has been found that the Arabs do not readily or willingly take advantage of these institutions. Therefore several small hospitals have sprung up throughout the country for the use of Arabs alone. Last year 15 of these hospitals were working, five have been opened since, eight others are in course of construction, and no fewer than 27 more are in consideration. These hospitals are under the direction of the communal or parish doctor (a European, of course), but the nursing is done by native men or women, and the cooking is in charge of an Arab cook, and is done according to the Mahomedan ideas and prescriptions. For patients not needing to become in-patients there are free consultations, which are held sometimes in the hospitals, at others on the market-places of the Arab villages. These efforts towards ameliorating the condition of the natives were found to be counteracted in a great measure, as regards women at least, by the well-known severity of the Mahomedan laws regarding the fair sex.

### Treating the Sick Women.

An admirable mission was therefore started some years ago by Mme. Chellier among the various tribes and villages. It remained for another large-hearted Frenchwoman to extend this charitable work and establish it on a firmer and broader basis. The ignorance of the natives on the most elementary laws of hygiene is profound; the sick generally owing their recovery solely to the original robustness of their constitution. Mme. Legey, doctor of medicine, conceived the idea of herself penetrating into the Arab circles, attending sick women, and giving advice to all. In spite of difficulties and much hard work, the attempt has prospered and developed. For some years the small hospital which she opened was confined to a gloomy little house in one of the narrowest streets of the Arab town. In November, 1903, a larger and more commodious house was acquired in the Rue Porte-Neuve, in a higher and more salubrious situation. The French tricolour over the doorway alone distinguishes it from the other Moorish houses of the neighbourhood.

### An Afternoon's Work.

One day I paid a visit to this hospital during the hour that Mme. Legey attends there for her afternoon consultations. By a staircase lined with bright-coloured tiles we reached the interior court of the first floor, where about 50 women, seated on straw mats and chattering together, awaited their friend and doctor. On her entrance they bowed and muttered words of welcome. From the court Mme. Legey entered her consulting room, well lighted by a large bow window thrown out through the ancient wall of the house. On the floor, crouching on mats, other women—the first comers—were waiting patiently. Before a little desk

### TENDING SICK NATIVES IN ALGERIA—*Continued.*

Mlle. Rogerel, a certificated midwife of the school of medicine and house doctor of the hospital, was seated. Two native women attended as nurses. The consultations went on apace. The women, with great volubility and excited gestures, described their symptoms and listened attentively to the instructions given to them. There are usually from 90 to 100 out-patients daily. The morning hours are reserved for diseases of the eye, which are so common among the Arabs. Friday morning is set apart for diseases of women. On Monday, Wednesday, and Friday afternoons, after one o'clock, the general consultation is held, when nearly 100 women come daily to seek advice.

### A Description of the Wards.

On the second floor are two small wards with 10 beds for serious cases. The rooms are bright and clean, simply furnished with low narrow beds and straw mats. In a recess of the wall is a long shelf pierced with holes in which stand basins and ewers, a simple but sufficient washstand. There are besides two accouchement wards of one bed each, with a table for minor surgical operations, and a bath-room where in-patients have to undergo summary ablutions upon their entrance. A small linen and store room answers also as a pharmacy. Here two registers of cases are regularly kept. We mounted next to the terrace roof, from which we admired the world-renowned view of the beautiful bay of Algiers. On one side of the roof the kitchen is built, where Yamina, an Arab woman, was preparing the evening meal on four small charcoal fires. The cooking is of course "à la mode Arabe" to suit the inmates. We saw the mênus for each day. This was Friday's: Morning—meat soup and couscouss (a preparation of semolina much in favour among the Arabs); evening—soup, meat and celery in sauce. On the other side of the roof is the house doctor's room, the windows overlooking a veritable sea of red roofs and white terraces. Such is the organisation of the new hospital for native women, whose growing success will probably soon necessitate an even further enlargement. The hospital is subsidised by the Government, who have also placed a certain yearly sum in the hands of Mme. Legey for the relief of necessitous cases under her care.

### Women's Dispensaries.

Free dispensaries and consulting-rooms for women, under the direction of lady doctors, have been opened at Algiers, Constantine, Bone, Oran, and Tlemçen. These are all well attended and much appreciated, and will doubtless be rapidly multiplied. The nursing in the small native hospitals leaves much to be desired according to our English ideas, as there are no really properly trained nurses. But the fact of being attended by one of their own people means a great deal to these poor women and compensates no doubt for the want of more skilled nursing. The constant supervision, too, of the house doctor and the daily visits of the consulting physician ensures the more essential part of the treatment being carried out, and many a poor woman must already have had abundant cause for gratitude.

### Beyond the Seas: Port Augusta Hospital, South Australia.

BY ONE OF THE STAFF NURSES.

Our hospital stands on a large sandhill, just two miles from the township of Port Augusta, which lies at the top of Spencer's Gulf. Here we seem to get more wind and dust than our share. In the summer the dust blows up dreadfully coarse red sand all day and all night, and in winter the wind shrieks and howls around most dismally. Of course we get some nice weather, and the warm sunshiny days are glorious. As we are just off the beach we have full view of the Gulf.

### The Staff and Wards.

Our staff consists of sister, three charge nurses, and three probationers, the charge nurses taking night duty for a month at a time. The night nurse is rather lonely, but there is always plenty to do, for there are over 50 beds. When all are filled we are very busy. Our wards are nice and airy, overlooking the sea, consisting of male surgical, male medical, three small fever wards, and an eye ward on the male side, whilst the female ward is alone on the other side; unfortunately, we are obliged to mix the surgical and medical female patients, having no other wards. With the padded ward, single ward, and cottage for isolation cases, the hospital is complete. It is very often filled with weary "sundowners," fever and all kinds and conditions of disease, and people too; for there are amongst our patients aboriginals, half-castes, Indians, and Afghans, and very good patients they are too. We have a great number of children, chiefly ophthalmic cases, especially in the summer.

### Our Few Excitements.

As the hospital is so far away from the town we do not get much excitement, unless the ambulance drives up, which happens constantly. Another excitement, especially for the children, is the passing of the weekly steamer; it is the signal for a stampede of the youngsters, adults, and the staff as well to the balcony, armed with sheets, table-cloths, locker-covers, towels and red handkerchiefs mounted on bamboo sticks, to wave to the passing boat. When she comes abreast the captain always blows his whistle, and he and all on board wave. We all respond with our washing. One of our friends, a captain of a large steamer—for they all have learned that we expect a whistle, even the strangers—gave us a very nice megaphone, and now we can talk to them as they pass along.

### A Visit from His Excellency.

We had a visit lately from his Excellency Sir George Le Hunt and party. They came round in his Majesty's ship *Protector*. The children quite expected the war boat to whistle and dip her flag as all the other boats, but to their disgust it steamed on with only a wave from some one on board. Our wards were decorated prettily and the balconies gay with bunting, and over the gate was a large archway of green in the centre of which hung the good old flag—the Union Jack. His Excellency and party inspected all wards, shaking hands with each patient; one, an old daddy of 86 years, clasped both hands round the Governor's hand, crying out "God bless you" several times.

### The Breach of Etiquette.

As the party entered the male surgical ward they were greeted by the National Anthem, sung by a little black boy with a fractured femur; he was rather shy at starting but finished very well. As the captain of his Majesty's ship *Protector* was one of the visitors, we complained to him of the breach of etiquette in allowing his boat to come in without whistling, cautioning him if the same offence were to occur we would certainly report him to the Marine Board! He apologised most profusely, promising us he would see that it should not occur again. We had no cause to complain, for when the war boat on leaving came abreast of the hospital a shrill unearthly whistle rent the air, while everyone aboard waved frantically, which was returned by all at our institution who were able to crawl out to the balconies.

### District Nursing in Holland.

### CHAT WITH A DUTCH NURSE.

"Can you give me an introduction to a district nurse who speaks English?" I asked the matron of the Haarlem Hospital while calling upon her one day recently. "Why, yes, certainly," she answered kindly. Then, producing one of her visiting cards, she wrote upon it "Luster Salm, Schagchelstr 17," handed the card to me and told me that if I liked one of the hospital porters should escort me there. I shortly afterwards sallied forth with the attendant.

It was a glorious afternoon, and as we left the gates of the hospital and found the sun pouring down upon the brick-paved roads and quaint old houses of brick and hewn stone on the one side, I felt the charm of the cool-looking canal and green trees on the other. However, we had soon to turn from the canal and trees and wend our way down a clean street, and one which, but for the strains proceeding from a Salvation Army hall, might also have been called quiet. At last my guide pointed to a nice-looking house, and there, on a large printed card in the window, was the name "Luster Salm." But, upon inquiring for her, we were told that she was out and might not be back for an hour. Would I come inside and wait? I particularly wanted to see her, so, dismissing the porter, stepped indoors.

I was shown into a comfortable-looking room of a burger house. Curiously enough, as I sat down, the first thing that caught my eye was a copy of The Hospital lying on a table. The atmosphere of the room was pleasant, and I did not grudge the time spent waiting there. The Dutch love of art, as well as comfort in the home, was everywhere apparent. Haarlem has many sixteenth and seventeenth-century houses, and this room was evidently very old. It had a beautiful old carved door, and a handsome antique fireplace and mantelpiece. With regard to the furniture, besides some comfortable chairs, an old bureau and a large inlaid chest made me very covetous. There was a good deal of Delft china about, and many bright silver and brass "bits"; also some curious old prints hanging on the walls. There were plenty of flowers, and plenty of books in several languages. Among the latter I noticed "Vanity Fair," "Aurora Leigh," and "Robert Elsmere." Altogether, many evidences of a refined, broad-minded gentlewoman pervaded.

At length Luster Salm came in, and, after a few mutual explanations, we began to discuss her work. "Yes," she said, "I have been nursing for seven years, always district nursing, and always in Haarlem. I was trained at the Wilhelmina Gasthuis, Amsterdam, and was there for three years, whence I emerged a fully-trained nurse, holding the White Cross certificate, and qualified in monthly nursing.

"Luster" Salm went on to tell me that she is attached to the Baptist Church in Haarlem, and that each church has its own nurse. Her district work seemed to consist of very much the same kind of work that falls to the average district nurse anywhere. She works under a doctor. She is not allowed to attend any infectious case or to sit up with patients at night. A great deal of her work is monthly nursing. She only attends to the patients, and where there is no one forthcoming to keep a sick person's room in order, she is allowed to provide a woman to do so. And although Luster Salm has no very poor people in her district, she is permitted by the church committee to supply her patients with many supplementary appliances and comforts.

By eight or half-past eight she is off every morning, either walking or on her bicycle. After her early rounds she has daily to be in attendance upon the church school-children in an out-patient room near the school. Then in the afternoons she has, when necessary, to go on her rounds again; while in the evenings she generally does some more visiting. She has also to attend some old folks in almshouses connected with the church. So her life seems most interesting and varied, and a very full one.

When I spoke of leaving, Luster Salm asked me if I would like to see her school-children's out-patient room, and an almshouse on my way to the station; at the same time she most kindly offered to accompany me to both places. Before we left her lodgings she gratified my curiosity by unlocking her old chest and showing me a wealth of beautiful old Delft china.

When we reached the children's out-patient room I observed that the room was bright, well-ventilated and lighted, and scrupulously clean. It seems that the school-children have to go there daily to be inspected by Luster Salm, and she attends to any of their minor ailments. The nature of those ailments can easily be imagined, for they are of the ordinary "school" order—heads, eyes, noses, or throats, chiefly. The room is fitted with a large bath, washing basins, a bed, a couch, and a cupboard for dressings. There is also another cupboard full of toys. "Yes," said Luster, "we keep toys to amuse the children while they are waiting to be examined. And if you are interested in things of that kind, see here"—and she pointed to a beautiful model of a ward in the Wilhelmina Gasthuis, Amsterdam. It was perfect, from the box-like beds to the white crosses at the nurses' throats. Before leaving the room I noticed a formidable-looking notice hanging up, and asked the Luster to be good enough to translate it for me. It was to this effect: "Notice to children! Water is cheap, therefore there is no excuse to be dirty. Water costs nothing."

Then from the modern *crèche* we went to an old almshouse. Holland is famous for its benevolent institutions, and Haarlem, in particular, for its *hofjes* (almshouses). Luster Salm led me up a narrow alley till we reached an ancient gate. She lifted the latch, and we walked into a courtyard. In the middle of the courtyard was a large bed of old-fashioned flowers, and all around were the little cottages. Luster knocked at one of the doors, and we were admitted by a former patient of hers, an old woman of 91 whom she had nursed from time to time. Though small and very wizen the old woman was quite active and most intelligent. Her tiny dwelling, a typical Dutch home in miniature—with its antique furniture, bits of old China and bric-à-brac, cupboard-bed against the wall, and portrait of the young Queen Wilhelmina—impressed me very much; while her welcome to the nurse, and kindly greeting to me, were charming.

. . . . . . . . .

And, dear little old Dutch woman, when later you pattered out of your nutshell home, and insisted upon opening the courtyard gate for us, I looked with added interest at the "Luster" and suddenly realised, meagrely, the breadth of her district.

### Nursing in the Isolation Block of an Irish Union.

It has been my fortune, together with another nurse from my own Infirmary, to spend a few months in the isolation block of an Irish Union. Our nominal superiors were a Matron and a Night Superintendent; in reality, everybody was ruled by a black-and-tan terrier and a feathered autocrat in the shape of a shrill-voiced parrot. Anywhere else these animals would have been treated as pets; here they were household gods whose high priests were the Matron and the Night Superintendent, and on whose shrines a daily sacrifice was made of our comfort. Our sitting-room was a small apartment, made cosy by a bright fire, which, however, often had the effect of rendering the atmosphere stifling. Naturally we had recourse to the window (near which stood the parrot's cage). We opened it widely, and were enjoying the pleasant mixture of cool air and cheerful fire, when the door opened and in walked the Matron! At first the window passed unnoticed, for the blind was down; but our unlucky star was surely shining that night, for suddenly she felt the breeze, walked over to the offending window, closed it with a bang, and, utterly regardless of our feelings, announced that it was never to be opened "lest Cæsar"—such was the weird name of the parrot—"should catch cold." This was bad enough; but when, a few nights later, we were forbidden the use of a second gas-jet, "because gas is so injurious to birds," we held a consultation as to the relative merits of a dose of carbolic or a few whiffs of chloroform as a means of lessening by one the number of parrots in the world. We were unwilling to inflict pain upon the innocent cause of our troubles, and therefore negatived the use of carbolic, whilst the difficulty of obtaining the chloroform prevented our choosing that more merciful method of destruction, so we had to make the best of an unhealthy atmosphere and insufficient light, and relieve our feelings by language which was "frequent and painful and free."

### The Subject of "Deputies."

The maids, or "deputies" as they were called, were an original set of women. On some points they seemed invested with an authority far surpassing ours, and often did the laundry-girl try to make our ideas coincide with hers on the subject of sheets, etc., which she doled out grudgingly to us. "Ellen," said one of us, the morning after our arrival, "bring me a fresh sheet, please." Ellen looked first at the bed, then at the patient; finally, her eyes rested reproachfully upon the nurse. "Shure, miss," she ejaculated, "he got a clane sheet a week ago, and besides," she added in an explanatory tone, "he'll be goin' out in a few days," as if that prospect covered a multitude of sins. Will we ever forget the feelings with which we faced our first breakfast, when our deputy, having placed the teapot and a pair of boiled eggs upon the table, departed with the cheering remark: "I hopes the tay isn't too sthrong, Miss, and the eggs is good—when they're fresh!"

### The First Sunday Morning.

I think that, even if I live to be an old, old woman, one picture will always retain its place in the gallery of my memory—a picture of the lofty, somewhat bare, church as I saw it on my first Sunday morning at the Union, crowded with rows upon rows of men and women, some very, very old, others pitifully young, but all alike in bearing the half-degraded, half-bold, and defiant look of paupers. I could not pray; my mind and my heart had room only for an overwhelming flood of pity for these my fellow-creatures, so many of them bending their white heads beneath the weight of their lonely and cheerless life, so many again missing all the love and innocent pleasures that make childhood and youth beautiful. About a week before St. Patrick's Day my deputy came to me with a box of shamrock, and, with many apologies, asked me to address it to her sister in America. How gladly I did so! for I thought of all the happiness that tiny green box and its bunch of shamrock would bring to the Irish girl in New York, and I could imagine the welcome it would receive after its journey as its leaves revived, and each gave its message of love and greeting from the far-off home.

### The Butter Supply.

Our experiences certainly ranged "from grave to gay, from lively to severe," and some of our heartiest laughs were at our own expense, as on one occasion when we found that the ideas of the Board had not quite coincided with ours on the subject of our butter supply, with the result that we came on Thursday to the end of our allowance, given out on Saturday. We gazed at the table with its empty butter-plate, then made a simultaneous voyage of discovery over to the sideboard. Like the Mother Hubbard cupboard of our childish days, it was bare! We looked at each other in silence; at such moments feeling is too deep for words. Then I volunteered to approach the cook on the matter. "Julia," said I, sweetly, "we've no butter." "Well, my dear," said she, equally sweetly, "I can't help that; your fresh buther won't be up from the Stores till Saturday." Visions of meals of dry bread rose before me, and I was busy "thinking" when she proposed, "I could give yez some salt butther, if yez would eat that." Little did I know what I was letting myself in for when I accepted her offer! Her ample form disappeared into the pantry, whence she emerged in a few minutes, bearing a plate of—well, for want of a better name, I suppose I must call it butter. I returned with my spoil to the dining-room, where we sniffed, ate one mouthful each of bread and butter, and . . . finished with dry bread and sugar. Afterwards we opened the window, thinking that perhaps the "butther" might find its way out unaided, and also to refresh the atmosphere. We augmented our supplies ourselves in future.

### The Value of Cheerful Companionship.

Looking back on those months spent in the fever hospital, I cannot but think of what they might have been with another fellow-worker. I feel that I owe a deep debt of gratitude to the nurse who was with me for her unfailingly cheerful companionship, her ever-ready help, and her sense of humour. It was a lesson to us both to realise how much patient suffering is to be found within the walls of one of these human beehives. Many of its inmates, it is true, have played the coward in the battle of life, but they have men's and women's souls all the same, full of passionate longing for what happiness life can offer, and men's and women's hearts beat none the less strongly beneath those brown coats and those faded shawls because the world forgets those who wear them, except to call them paupers! Our lives as nurses seem to be made up of scenes of deep pathos and incidents the humour of which lingers long in our memory, and nowhere, I think, are these two extremes more perfectly blended than in a Union such as that which was our abiding-place for half a year.

### The Evolution of an Island Hospital in the Pacific.

### HOW IT WAS DONE.

It was a most unpretentious-looking building, not in the least to be compared with the hospitals of England, save in this, that, like them, its aim was to help and to save suffering humanity. It had at one time been a warehouse, and would, in all probability, have long since been pulled down to make room for the erection of a fine branch store by its owners, a well-established trading firm, but times had been bad, money scarce, business slack, and so the old warehouse had held its own until the heart of the ruling magistrate of the island conceived the idea of acquiring it and its grounds—very circumscribed—and turning it into a cottage hospital. The thought was altogether new: hitherto no abode had been set apart in that island for the sick, and it remained to be proved whether the innovation would meet with success among the Maoris, or whether the strangeness to them of the arrangement, coupled with a still lingering dislike to the "white man's physic," would make the scheme a failure.

### The Managers.

At first its management was a so-called "Government affair," medical officer and nurse being directly appointed by the chief magistrate. Soon, however, there appeared in the minute book of the Island Parliament the following entry:—"Whereas it is desirable that the hospital should as much as possible be under the supervision of the people, and receive their full sympathy and support, be it enacted, &c., &c.," and authorisation followed for the establishment of a hospital board, to consist of 12 members, chosen in equal proportion from whites and Maoris, and since, in the case of whites, women were considered "eligible for election," and because of "experience gained among the sick in another island," I had the honour of being put upon this board and installed as treasurer. The doctor at this juncture in office was a Seventh Day Adventist, the nurse, too, was of similar religious persuasion, and could trace her descent directly back to the mutineers of H.M.S. *Bounty*.

### Various Dilemmas.

I suppose that hospital boards in England sometimes have difficult matters to deal with, and I know the question of finance is to them very often a puzzling one. Such, too, was our experience out in the Pacific. Possibly it may also happen at home that a medical man diagnoses wrongly. Here, then, was our dilemma: funds limited, a case grievously misunderstood, and a deliberate attempt to proselytise. How well I remember the somewhat stormy interview I had with the doctor on this last-named point. It did seem abominably unjust that a very sick patient should be tortured with all the intricacies of sectarian differentiation. Anyway, it was a decided breach of covenant, for the hospital was to be unsectarian, open to all faiths and no faiths. Of course we decided, as a board, that the medical officer must go, and due notice was given. It so happened that news of the impending change leaked out just as the monthly steamer was leaving. A certain passenger happened to be going on to San Francisco, and *en route* he chanced to come across two young Scottish doctors, to whom he mentioned what he had heard concerning our hospital. They at once determined to come and see what the approaching vacant berth was like, deeming it a possibly good opportunity to gain some practical knowledge of tropical diseases. They came, they saw, they conquered, and were conquered.

### The New Medical Officers.

The advent of these young Scottish doctors ultimately led to the hoisting of the British flag over one of the fairest spots throughout the length and breadth of the Pacific. Pity rather than condemnation should, I think, be given to those who conceive good and great ideas, but who, owing to some defect—mental, moral, or physical—or possibly to a combination of all three, are not fitted to carry the ideas into realisation. It would be unwise, perhaps, to attempt to go into all the details that eventually culminated in a change of Government. History was in process of making; the writing of it out in full is possibly better left to a later generation, who, viewing in right perspective, will, perchance, be better able to adjudge blame and praise. To those of us who were in the very midst of the turmoil, the heart-burning, and the change, the time was one of great excitement, and could the wooden walls of that little cottage hospital by the sea but receive the power of speech, what tales of interest and wonder would they disclose! Amid it all the work of healing went on. The new medical officers proved themselves men of ability and skill, and possessed of the power to win the confidence of the native. They had to "rough it" at the first, but "roughing it" is calculated to bring into evidence and use the grit that is in a man's nature, and it is just possible that, great as was the amount of good these doctors did, the benefit they received equalled, if not exceeded, it.

### Economy and Episodes.

The funds for all working expenses were very limited, and great economy had to be practised; native appliances had to be turned to account, wherever possible, and native help trained and utilised. Occasionally amusing episodes would arise; occasionally, too, some very proper people would receive a shock. I remember causing consternation once because I donned a capacious apron, turned up my sleeves and helped to clean the windows. Instruction by word had failed to obtain from a native the kind of clean windows I deemed desirable in a hospital, so I thought I would just clean them with the boy for once. Ah me! the surprise and remonstrance that it brought forth! However, the deed had accomplished what was aimed at; henceforth the windows satisfied me. Sometimes patients caused trouble by persistently recurring to native medicine and native doctors. In one special case, death resulted; in another, life was saved almost miraculously, certainly by the exercise of exceedingly prompt and well-directed skill. An epidemic of influenza had reigned; many cases had been cured, some deaths had occurred. I had been fortunate with those committed to my more especial care; every one had recovered. Then my caretaker's wife sickened. Finding that she was becoming very ill, I made arrangements for her admission into the hospital. She was carefully wrapped up and conveyed in a comfortable buggy to the hospital door, and then refused, absolutely, to go in. The driver, a relative, took her to his own "European-made" house, and communicated with me. I at once interviewed the younger doctor—the brother was absent among some neighbouring islands visiting sick folk there—and he consented to treat her "at home." But she would neither obey orders nor take the "white man's physic," and managed somehow to get up and crawl away to a "native house" near by, like a wounded animal to its lair. There she grew steadily worse, and was at length transported back

THE EVOLUTION OF AN ISLAND HOSPITAL IN THE PACIFIC—*Continued.*

home to die, in the buggy which had at first taken her where in all probability she might have regained power to live.

A Contrast.

The other case was that of a wee laddie. He was out of my jurisdiction. Influenza seized hold of him. His relatives could have applied to me, but they did not. Native remedies were used, but apparently did no good, for when, by mere chance I happened to look in at their "hut," the child appeared alarmingly ill. I never have understood what the old native meant, but he murmured something that seemed to me dreadfully fatalistic. Fortunately, he trusted me personally, and when I expressed a desire to arrange for the boy to go to the hospital he consented. Very quickly action had to be taken, for the day was drawing to its close, and a drive of about three miles lay before the patient. Leaving the procuring of the buggy and the wrapping up of the child to those I could trust, I went on in advance in search of the doctor. Providentially he was not far from the hospital, and by the time the patient arrived the medical man was ready to receive him. British skill and promptitude averted what might have been disaster, and after a prolonged residence in the hospital, during which time many inches were added to his stature, the laddie left fully cured, but somewhat changed in appearance.

. . . . . . .

Weeks and months sped past, new faces appeared in the island, old faces disappeared, summoned to duty elsewhere; some ties were broken by circumstances or by death, new ties were formed; but amidst it all the hospital stood firm and steadfast; the days of turmoil ended, friction became a thing of the past, and Britain's flag having brought tranquillity, the wisdom of British rule was placed beyond dispute.

### Private Nursing in India.

BY AN ARMY SISTER.

It is not generally known that a large field for private nursing exists in India. If a woman, physically and mentally capable, could make up her mind to go bravely forward in this work, willing to meet and combat all the inconveniences that must of necessity arise, and, not disheartened with difficulties put her best work into it, the possibilities would astonish many.

The Equipment.

She would require money and some connection in the country. The money must comprise a good sum in hand, to be put aside for use in India before she can establish herself. Passage and outfit money, say £50, and for immediate expenses for a month until settled, about 300 rupees or £20. This would be ample, but only for a month. Her uniform need only consist of white drill skirts and some thin blouse bodices, linen aprons, caps, and six pairs of boots and shoes, and some underlinen; but the nurse must bear in mind that a dhirrgie or native tailor can for eight annas (8d.) a day, make well anything she may want from patterns. They can as a rule use a sewing machine, so it would be a good thing to take one out to India, as it would save much time and expense.

Introductions Wanted.

A nurse will, in addition to knowing friends, have to ensure that they, and those, in England are in a position to introduce her to medical men in India of good position and reputation. They, in their turn, would surely help her in such a good cause, and it will rest entirely with the nurse as to the success of her venture. She must be in earnest, and in all respects fitted for the post as regards education, tact, and ability. When once the doctors have trust and confidence in her, she will find more work than she can possibly perform. It will be uphill work for a time, but patience will assuredly win the day.

How to Start.

At first I would advise the nurse to go to a large civil and military station, and at once present her letters of introduction, calling also on the leading doctors of the place. Here tact will have to show itself, as there are many calls on medical men, and they, naturally, do not always feel ready to trust a stranger. Having made herself known, I should advise her to take two rooms as a boarder. This should not cost more than 3 or 4 rupees a day, or 90 rupees to 100 rupees per mensem. She need have no other expenses at first except a man-servant, or "boy," as he is called. He is useful in many ways—for cleaning and dusting the rooms and taking care of them in her absence, running messages, etc.—and his cost a month would be 8 or 10 rupees. Then there must be a time of anxiety, but it would not be wise for the nurse to fix her fee lower than 5 to 7 rupees a day, as unless she takes those cases who can pay there would be loss. But at the same time there are many subalterns' wives, or even captains' with perhaps large families, who require a good nurse immediately, though they could not afford to pay so much. In such cases the kind heart of the nurse must act as it dictates, and for the first few months a little money would be better than none at all. Amongst rich and influential families she would naturally get her own price, and I feel sure that a little consideration shown at the outset will reap its reward.

A Practical Suggestion.

I spoke of boarding in two rooms. This might be a difficulty, as perhaps the nurse would not find a suitable resting-place where she could be comfortable and have congenial surroundings; so that if it could possibly be arranged that friends could take her to live with them for a short time, paying them a small board, it would be better in all ways, as she would have more opportunities of informing herself in the ways and customs of the country, and of settling down with less anxiety, having friends at her back.

The Nurse Herself.

Now I would like to say a little more as to the nurse herself. She must be a gentlewoman, accustomed to move in good society, but with no love for its gaieties—in fact, proof against them. It must be work—and a resting time—for some months or a year or two till a measure of success is gained. Then, again, the nurse must be pleasant, bright, tactful, patient, and sweet-tempered through a good many adversities, prepared to take the bad time with the good, to be strong, enduring, and capable of standing a fair amount of fatigue in a trying climate, for, of course, the nursing is done in the tropics, and the hot months all over India are very trying. Is there one among your readers who is willing to try, and who answers to all that is required, both as regards character, temperament, and a little money? If so, let her be up and ready, for success will surely be the result of steadfastness, and the thanks and gratitude of many await her out here!

### English Nurses in a French Hospital.

It happened recently that two English nurses found themselves in the unusual position of nursing an English patient who was suffering from typhoid in a large French hospital. They were asked, on their return, if they would not give their experiences for the benefit of their comrades at home. They demurred to this, saying that they had met with so much real kindness at —— that they would not like to write anything that might seem to reflect on their hosts; and yet a good deal that they met with compared rather unfavourably with what is the rule in our own hospitals. However, here is a short account summarised from some of their letters.

### Nursing by Nuns.

The hospital is nursed by nuns of St. Augustine, an Order which has existed for the last 800 years, the Sisters wearing the same robe that was worn at its founding, with the exception that they left off the black veil at the time of the introduction of modern antiseptics. One of the nuns showed me how all their clothes were pinned on, with the exception of strings to their aprons. Their clothes are all made in one piece, and are all washing things. They admired the English aprons very much, as theirs are unstarched holland ones. When anyone applies for admission to the Order she is on probation for four months, and then becomes a novice. It is a *fête* day when they take the veil and begin another two years' probation; and during those two years they may leave at any time, if they do not feel a vocation for the work. But at the end of that time they "sign on" for the rest of their lives or leave. If they stay they give up everything, they never leave the hospital again, never have a holiday, and never have any off-duty time, except half-an-hour after their dinner in the evening. It is small wonder that they should be pale, but they all have a look of perfect happiness, and are always bright, and are devoted to their work. They get up at four or half-past, and go to bed at nine. The *sage-femme* was particularly kind to the nurses, and would often take them round different parts of the great buildings.

### A Strange Proposal.

On the nurses' arrival there was some difficulty about their sleeping accommodation. The patient was in a small typhoid ward with two beds in it, and it was arranged that the nurse not on duty should sleep in the second. Of course they could not agree to this, nor to a proposal that she should sleep in the bathroom, especially as this was used not only for bathing but for keeping soiled linen, and in the corner was a large bucket in which everything from two or three wards was kept till the morning, for there were no sanitary arrangements within doors. So the nurses found a little room in a small café near the front gates, where they also had their meals.

### The Unskilled Nurse at Work.

The English nurses found great interest in nursing under the French doctors, for many of their ways were utterly unlike those in which they had been trained. For instance, the patient, from her admission, had seven cold baths a day, walking from her bed to the bathroom. The other typhoid patients in the same block used to walk along the stone floors with bare feet! The baths were continued until the temperature was normal, when food of almost any kind, including beefsteak, fried potatoes, and strawberry jam, was allowed at the end of a week. The patients were not disturbed at night for food or even temperature, and generally slept well. There was only one nun on night duty, who was changed every night, and everything was done by the very unskilled women who worked under the nuns. These *bonnes* have no regular instruction in nursing, nor are they even supposed to do anything but the rough work. The consequence is that, as the sisters cannot do a quarter of what has to be done, the patients come off badly. One night the English nurse, going through the next ward to the bathroom, found that one of the two patients in the ward had just died. She was lying on the bed as she had died, her eyes open and jaw fallen, not even her face covered. There was no screen in the room, and the girl in the opposite bed, who was very ill and only sixteen, was terrified. But the *bonne* took no notice, and simply said that she could do nothing till the sister came on her next round in about two hours! Another *bonne* told the night nurse that a patient would not take her food. She had no idea of trying to make her take it. All the food is put on the lockers and if the patient is well enough to help herself to it, well and good, but, if not, she dies, as actually happened in this case. Another patient was delirious for a long time, and when she had recovered to some extent, the night nurse saw her sitting up in bed cutting off her hair. It had been allowed to become perfectly matted together during her illness, and the sister had told her to cut it off, and the girl did not object in the least. The general wards are large and bare. They have about 34 beds, in two long rows, with a chair at the bottom of each bed, and that is all; no pictures, no pretty tables, no couches nor easy chairs, and scarcely any flowers or plants. The food is very good, but quite different from ours. A great deal of soup is used.

### No Toys for the Children.

After they had made friends with the sisters, the nurses were taken by degrees all over the hospital. There was a large lying-in ward, with a resident lay midwife, a very clever woman, who scarcely ever called in a doctor, managing forceps, version, etc., herself. Here pupils were received for a course of two years' training, which costs 1,200 francs in this country town, though in Paris the course takes 3,000 francs (£120). The children's ward was painted a cheerful green and contained nice little beds, but there was not a single toy to be seen, and though many of the children were surgical cases, the quiet of the ward was astonishing. There were two babies with typhoid, each with a little bath at its bedside, ready for it to be lifted in. Most of the buildings were old, including the theatre, which boasted of only one tap, basin, and sink.

### The Lunatic House.

The kitchens and laundries are huge places, very different from English ones. The kitchen stove is in the middle of the room, and there are immense boilers for the soup. Adjoining the hospital proper are many other buildings, of which one is a crêche, and another one a house let out for private patients. The saddest sight there is the lunatic house. In that department alone there are 700 patients. The violent cases were all locked together in a long narrow room or corridor, more like a passage, where there is just enough room for a long narrow bench for them to sit on. Some were chained to the walls, others were sitting with their hands tied behind them. Of course they were all raving, and when the superintendent opened the door, it reminded one of the nurses of going into a cage. There was straw on the floor, and the smell was awful. The superintendent said that the buildings are very old, and would, he hoped, soon be replaced. He asked lots of questions about English treatment. In this French hospital they use cold baths and douches for many diseases, and even for violent insane persons. There was one woman who was sitting in a bath where she had been for seven hours. The baths are covered, and have a hole for the head. This poor woman looked half starved. Cupping, both wet and dry, is much employed in the wards, and is done by the nuns. They use dry cotton wool for the glasses. They give hypodermic injections almost always in the buttock, plunging the needle straight in with one hand, while in the other a wet swab is held. A curious arrangement is that of the mortuary chapel. It is situated near the main entrance of the hospital, close on the street. The body of each person who dies in the hospital is placed there, with candles at the head and foot, and the door left open. It is touching to see the passers-by cross themselves or stop a moment while they say a prayer for the dead. The English nurses were a great puzzle to the inhabitants at first, who would even stand still in the street to stare after them, but they never met with anything approaching to incivility. They were very sorry on their own account when the time came that their patient was convalescent enough to be taken home; for though they saw much that might have been better done with more skilful training, they cannot speak too highly of the kindness of the good nuns to themselves and to all with whom they had to do; and they came away also with a high opinion of the French doctors and their ways.

### Nursing Consumptives at the Royal National Hospital, Ventnor.

INTERVIEW WITH THE MATRON. BY OUR COMMISSIONER.

As I drove up to the entrance gate of the Royal National Hospital for Consumption, at Ventnor, and observed the gardens and woods bright with spring flowers, and the sea glittering in the distance, I came to the conclusion that if the charms of nature could bring back the glow of health to

An Outdoor Revolving Shelter for Open-air Treatment.

the cheeks of the inmates, every patient should quit the institution sound and strong. Reaching the porter's lodge, I declined the offer of my charioteer to drive in, because I believed that I had arrived at my destination. But, although the hospital is only a hundred yards or so removed from the road, it is a quarter of a mile in length, and the house in which the matron resides is nearly at the end. It is the boast of the institution that it is conducted on the separate system, and the truth of this assertion is very apparent. Every block is a little self-contained hospital, each dedicated to some benefactor, the first being named after Princess Louise, who laid the foundation-stone on behalf of the late Queen Victoria.

In block 9 I found the matron, Miss C. Stuart Cameron, n her pleasant sitting-room looking over the picturesque grounds of 22 acres, which stretch right away to the sea shore. I congratulated her upon the beauty of the surroundings.

"Yes," said Miss Cameron, "and I have not yet been here ong enough for my pleasure in the view to grow dim by familiarity, for this is my first spring in Ventnor."

"Where were you before then?"

"I was assistant matron at the City of London Hospital for Diseases of the Chest, Victoria Park, so that you can imagine Ventnor is a great change for me. I was trained at the Royal Infirmary, Dumfries, then I became sister at Cardiff Infirmary, and afterwards night superintendent at the Preston Royal Infirmary and assistant matron at the Sheffield Infirmary."

"Do you find the work here similar to that at Victoria Park?"

"In many ways it is very different. There most of our cases were acute, here comparatively few are in that stage. Out of the 155 patients—94 men and 61 women—there are seldom more than 25 confined to bed. Also the class of patients are not the same; those in London were mostly drawn from the very poor, here they are of a superior class. This is partly accounted for by the fact that each patient has to pay 10s. a week towards his or her support."

"How long do they remain at the hospital?"

"They are admitted for eight weeks in the first instance, but if desirable they are given an extension."

### The Staff.

"As the cases are not often critical the nursing, I suppose, is comparatively easy?"

"It is not so arduous as in the general hospitals, and therefore we are able to take probationers at an earlier age."

"How early?"

"A few start at 20, but the majority begin their training

### The Daily Routine.

"What is the daily nursing routine?"

"The probationers rise at six, have breakfast at 6.30, at which meal the night superintendent presides, and enter the wards at seven o'clock. At 12 dinner, at which I preside, is served; 3.30 to four o'clock is tea time, and 8.30 is the hour for supper."

"And what off time do the nurses get?"

"They are off duty for two hours in the morning or afternoon, and four hours on Sunday. It is compulsory that out of this time they attend one service in the chapel. They also now get one day a month. They have a yearly holiday of 16 days, and the sisters a month."

"Do you find that the nurses suffer at all from constant contact with such an infectious disease?"

South Side of one of the Blocks for Women Patients.

at 21. Then they remain here generally for a couple of years, though a few leave at the end of twelve months, and if suitable for general nursing I endeavour to get them into a hospital where they can go in for their three years' certificate."

"From what class are they drawn?"

"Mostly from the professional classes. It is specially desirable here that it should be so."

"How is your staff made up?"

"It consists of a night superintendent, six sisters, and 20 probationers. Each sister—they have all been trained in good schools before coming here—is responsible for two blocks, and each block has its own probationer and housemaid, and is quite distinct from the rest of the hospital."

"Surely it must be difficult for you to superintend everything when the hospital is so scattered?"

"Yes, it is. The greatest difficulty is the long distances to be traversed. To go from one end of the hospital to the other without entering a single room and to return means half a mile, so that much time is spent in walking about. There are 30 maids to look after in addition to nurses."

"No, not at all. They are all carefully examined when they come in by the doctor, and those who are not perfectl strong are not accepted, but after a short time I find they are generally the picture of health. In one of the blocks there is a bedroom with two beds in it which is kept for the nurses' sick quarters, but I am glad to say that it is seldom occupied."

### The Nurses' Quarters.

"You have no nursing home, then?"

"No; but doubtless in time we shall have one. At present the nurses have a dining-room in one of the blocks, but they have no sitting-room. The sisters, however, have a sitting-room of their own. The nurses and the housemaids each sleep in their own blocks. In this block we have as well as the dining-room, offices, my own rooms, the quarters for the three resident doctors and the chaplain, the bedrooms for the nurses and the housemaids, and the sleeping apartments of 18 patients.

"But could not the patients occupy the rooms the nurses vacate?"

"No, because all the patients' rooms—each, you know, has

### NURSING CONSUMPTIVES AT THE ROYAL NATIONAL HOSPITAL, VENTNOR—*Continued.*

a separate bedroom—face south, so that the sun and sea breezes may always come in at the open windows, all the staff occupying the rooms overlooking the road, which face north."

### Duties and Salaries of the Nurses.

"What are the principal duties of the nurses?"

"Taking temperatures and pulses daily, seeing that nourishment is consumed every two hours, looking after the patients that they take their rest before and after meals, and weighing the patients every week. In cases of acute illness, a special nurse is appointed for the patient. In every block there is a medicine-room downstairs, which is under the care of the sister in charge. We have also a lady dispenser."

"How long does a nurse remain on night duty?"

"Every nurse does three months' night duty under the night superintendent. Each bedroom is visited three times during the night. We pay our night superintendent £40, the sisters £30, and the probationers £10 per year. They are all provided with indoor uniform."

On my way back the matron took me all over the hospital. First we visited the delightful dining-room, where the tables were prettily set out for tea, with large palm ferns at regular ntervals, and even the butter arranged in dainty pats so as to tempt the appetites of the invalids; then on to the big kitchens; an ext to the chapel, where, I was told, there s service at 11 and 3 every Sunday, Holy Communion service wice a month, and Bible classes during the week. Emerging out on the terrace, I noticed the covered balconies which run the entire length of the hospital on the ground and on the first floor. Here the patients lie out on lounge chairs covered with warm rugs whenever the weather is at all suitable, and the French windows leading into the rooms are always open. The patients' bedrooms, with their "roomy" beds, their polished floors, dressing-table, and wardrobe, look very cosy, and the sitting-rooms have plenty of sofas and easy-chairs, as well as a billiard-board, games, etc. Between the pillars of the verandahs, hammocks are swung. The men, as well as the women, have a croquet-lawn for their use, and they are allowed to receive friends every afternoon.

### The Two Lamps.

I also inspected one of the revolving shelters, which can be swung round so as to completely shut off a cold wind, and enable the patients to be out in the grounds even in chilly weather. Upon our return we went by way of the subterranean passage which runs the full length of the hospital. It is this passage the night-nurses traverse when on their nocturnal visits. The sister carries a red lamp, the probationer a green one. When either passes up the staircase into the block above, she leaves her lamp in the middle of the floor of the passage opposite the stairs, so that anyone suddenly needing the sister or probationer and going into the subway to find her can see at a glance in which block she is at work. With an involuntary smile as I recalled to mind the well-known "Lady of the Lamp," whose progress through the crowded wards of a Crimean hospital was so different to that of the night nurses through the lonely passages of a well-organised institution, we arrived once more in the open air.

### The Russian Red Cross Society and its Sisters.

In 1854 the Grand Duchess Helen sent at her own expense to Sebastopol the nurses whom she had assembled under the name of "Dames de l'Exaltation de la Croix" which brought together for charitable work Catholics, Protestants and Orthodox. Officially, however, the Red Cross Society was not founded in Russia until 1867, when it was placed under the patronage of the Empress. An earlier date is also given for its foundation—viz. in 1860 at Moscow. The Russian Red Cross Society is reported to be therichest in the world. Its resources are described as inexhaustible, and its expenditure, since its creation, more than 420 millions of francs. In 1896 it had paid out for sanitary work 1,537,000 roubles (the rouble being worth about 2s. 10d. of English money). In 1897, 1,573,997 roubles. In 1898, 1,107,975 roubles, and in 1899, 1,601,512 roubles. Services have been rendered by the Russian Red Cross in War—in the expedition sent by the Government of Russia into Turkestan in 1868; the expedition to Kuldja and Ourga, 1871; the Franco-German War, 1870–71; the Montenegrin Turkish conflict, 1876; the Servio-Turkish War, 1876; the Turkish War, 1877–78; the expedition against the Akhal Teke tribes, 1879–81; the war between China and Japan, 1894; the war between Abyssinia and Italy, 1896; the Græco-Turkish War, 1897; the Transvaal War, 1899; and the expedition to China, 1900.

### The Present Campaign.

In the present campaign it is playing a rôle of great magnitude. Since the commencement of the present hostilities the Society has mobilised 26 ambulances containing 5,500 beds, the executive committee being presided over by Count Vorontzoff Dashkoff. It possesses 42 hospitals, two schools for nurses, eight asylums for disabled soldiers, an asylum for widows, four homes for sick soldiers' children, and two sanatoria for children. It employs communities of sisters of charity numbering more than 4,000. These sisters are not under religious vows. They receive a training which lasts a year, some going through a course extending over three years. In 1897 the Society formed a body of male nurses—the Brethren of the Red Cross—which did useful work in China.

### The Society's Headquarters.

The establishments possessed by the Society at St. Petersburg are well warmed, well ventilated, and thoroughly lighted by windows. The walls and ceilings of the rooms and corridors, white in colour, give an agreeable appearance. Everywhere minute cleanliness and perfect order reign. There are operation-rooms, laboratories, disinfecting and sterilising chambers, dispensaries, etc., all well organised and in accordance with the most rigorous hygienic requirements. To the hospital, into which medical and surgical cases are admitted for treatment, is attached a dispensary, where every day a crowd of people of all ages come to consult the doctors and receive medicines and prescriptions.

### The Work of the Sisters.

A few figures will permit of a judgment being formed on this point. In the year 1900 some 1,020 sick were treated by the community of the Sisters of St. George; of these 141 were surgical patients and 108 operation cases. At the dispensary there were 225,999 consultations with medicines prescribed. The Russian Red Cross Society besides granting an annual pension of 250 roubles to sisters having 25 years' service, has founded for their benefit a "participation fund" or bank. Moreover, retired sisters can always avail themselves of asylums founded especially for them at St. Petersburg, Moscow, and Tiflis.

THE LOCAL COMMITTEE.

The Society gives unusual facilities to all those who desire to work under its guidance. If there be five willing men animated with a desire to serve it in a town or in a village this number is sufficient to form a committee. Such a committee has to be constituted with the consent of the Governor of the Province. It communicates with the local office of the Society or with the central committee in St. Petersburg. It is subject to all the formalities of the Red Cross Society. If it is desired to form a local committee in any chief or county town or headquarters, at least 30 persons must show their willingness to become its members.

THE UBIQUITY OF THE SISTERS.

Actually, there is, so to speak, scarcely a single province, not alone in Russia in Europe, but also in Russia in Asia, in which there does not exist some community of Sisters of Charity of the Red Cross. They were even in Port Arthur. Sick nurses of the Red Cross Society are chosen from among the young female pupils in the Imperial Schools. When they have completed their studies they are distributed among the military hospitals in numbers varying according to the importance of these establishments, *e.g.* 20 at Warsaw 17 at Moscow, three at Nijni Novgorod. During a campaign they may be found accompanying convoys of wounded or in sanitary trains destined for the removal of sick. A little previous to the present outbreak of hostilities the Society sent about 100 nurses to Manchuria to organise its ambulances and to complete the personnel of the army hospitals.

THE FINANCIAL CONDITION.

The latest account of the financial condition of the Russian Red Cross Society appears in the *Sviet* of June last. The total sum then in hand was £737,087. For the special account of the Russo-Japanese war it had received £356,222, and paid out £298,925 ; the balance left, less a fraction, was £57,296. Besides this there is a reserve capital for the pressure of war time of £227,951. The May receipts were £117,341, and the payments were £82,756.

### Incidents in a Nurse's Life.

### HOW WE MANAGED IN AN EMERGENCY.

**Contributions for this column should be addressed to the Editor, and if accepted will be paid for.**

THE baby was screaming, not with temper, as hard-hearted people would suggest, but with genuine pain. He was only ten months old, and suffering agony. His mother called me into the nursery. "Aunty Peggy," she said, "do come here; baby's ill." I (as is usually the case with maiden aunts) answered with all the wisdom of a great-grandmother, "It's stomach-ache, I expect, dear. Let's bring him near the fire and apply hot flannels." We did so; but still he continued to utter short, sharp, piercing screams, which went through both of us.

Baby was getting "ashen" now, and his face looked quite old and pinched; moreover, he was passing blood from the bowels. I had not wished to frighten his mother, but this last symptom decided me. I got my hat and ran for that almost sacred being (in the eyes of mothers) the "doctor." Of course I told the doctor as well as I could the nature of baby's screams, his looks, and the passing of blood. "My assistant and I," he answered, "were on our way to a house near yours, but," he added, turning to his assistant, "we will look in at Mrs. R——'s first." It was fortunate they did. As soon as the doctor had examined the child he pronounced it to be "intussusception," adding: "What we can do must be done at once." In a few minutes we had the nursery turned into a tiny theatre. We drew the table near the light, spread a mackintosh and blanket on, got a towel and an enamelled bowl in case of vomiting, and a large handkerchief folded over several times for the chloroform.

"Have you a bicycle pump in the house, Mrs. R——?" the doctor asked. "Yes, I'll fetch it," I answered. Mrs. R—— looked very indignant. What had bicycle pumps to do with her baby's sickness?

Of course, nurses will know its use. The doctor with its aid inflated air into the bowels until the protruding part of the intestine had slipped back into its place.

Meanwhile I ran and made up a bed on the nursery sofa, putting hot bottles in; and in about ten minutes' time the little patient was put into it. Our orders then were to diet him carefully and keep him warm. So in about three days' time, owing to the doctor's promptitude and the careful carrying out of his orders, baby was himself. Intussusception had been caused by indigestion and constipation.

### Select Committee of the House of Commons on Nursing.

EVIDENCE OF MISS FORREST.

MISS FORREST, proprietress of the Victoria and Bournemouth Nursing Institution and Home Hospital, formerly sister at Guy's Hospital, and matron of York County Hospital, was the first witness before the Select Committee on Wednesday. Her evidence was wholly in favour of State Registration on the grounds of the difficulty of discovering what knowledge was possessed by individual nurses, owing to the different standards prevailing in different hospitals, and even in the same hospital at different times (owing to changes among officials); the absolute want of standard in education, and the impossibility of tracing a nurse, owing to no records being kept. In reply to questions, Miss Forrest said that she had four houses at Bournemouth, and employed 160 nurses, some of whom were salaried, while others worked on the co-operative principle, paying a percentage on their fees. She took great pains to investigate all applications, and out of 460 who applied in 1904 was only able to engage 65, although she took all those who proved to be eligible. Length of training was not so much a difficulty, the essential point being the character of the instruction given in the time, and this varied with each matron. At the same time she regarded three years as the minimum for safety. She confessed to having suffered "agonies" herself through inadequate early training. She did not think more than three years necessary; this in a hospital with 50 beds should be sufficient. On the question of an examination conducted by a central body, Miss Forrest was of opinion that this should be partly written and partly clinical, and, on the suggestion of the Chairman, said that she did not think it would be possible for a candidate to deceive her in regard to practical knowledge tested by this means. The question of moral fitness must be dealt with by the hospital where each nurse was trained, the matron's certificate being the criterion. A member of the Committee having suggested that this would have the effect of vesting entire authority with the matron, Miss Forrest said the House Committee would be bound to see that no injustice was done, and the certificate could not be withheld without their knowledge.

On the Chairman's suggestion that the House Committee would be, to a great extent, in the hands of the matron, Miss Forrest said the matter could be referred to the medical staff.

On the question of the proposed register, Miss Forrest thought a three years' revision would be sufficient, provided that the nurse reported herself at stated intervals. She was not prepared to enter into the details of finance, but her opinion was that a fee of one guinea for the examination would be sufficient. With regard to the removal of names from the register, loss of moral character, and, in certain instances, loss of efficiency, must be the determining causes, both being fully substantiated. She was entirely of opinion that the registration and inspection of nursing homes would be an excellent thing, there being undoubtedly abuses which required drastic treatment. She did not think registration would greatly affect the supply of nurses one way or the other. Questioned by the chairman as to the proposed registration of two classes, with a lower and a higher standard, the witness, while not admitting any fundamental objection to the proposal, held that it would lead to confusion in the minds of the public, who would not be able to distinguish between a fully-trained nurse and one qualified to carry out the duties of a " nurse-help " in rural districts. Sir John Batty Tuke objected to the phrase (used by the chairman to describe a certain type of village nurse) and said these women performed the duties of a nurse. Miss Forrest did not think a highly trained woman should cook another person's dinner; it would be simpler not to include this class in the register. A member of the Committee having made a comparison between nurses in England and dentists in Germany, when he was informed there were two classes of the latter, Miss Forrest suggested that the difference in the uniform might, in the case of Germany, differentiate one dentist from another. She regretted that in Bournemouth there were half-trained women competing with those who were fully trained; the public was deceived by the word " certificated," which might refer only to a monthly or other special course. They besieged the doctors, and in some cases adopted the uniform peculiar to her own institution.

Evidence of Miss Wortabet.

After the Committee had adjourned for half-an-hour, Miss Wortabet was called, her evidence occupying only half-an-hour. Having explained that she had been a paying probationer at the London Temperance and the Middlesex Hospitals, the witness said she had held the position of lady superintendent of St. George's Hospital, Beyrout, where she not only trained Syrian women as nurses, but wrote a book on nursing as adapted to the needs of the East. She had also had experience in Egypt, and in France, at Paris and Bordeaux. She was emphatically in favour of State registration as a remedy for the diversity of training in the various hospitals at different times and even in different wards. The same standard must be insisted on for the poor as for the rich; the work of nursing should be raised to the dignity of a profession, and the better educated a nurse was the better her work would be; although it was not a question of money, the services of a highly-qualified woman ought to be adequately recognised; she herself, after spending nearly £300 on her training, was about to take a country district for £45 a year. Nurses should sacrifice something for the privilege of being on a State Register, and she did not think three or even five guineas too much as an examination fee. Though the work should be in the hands of educated women, with a high standard of attainment, a yet higher altitude should be reached by those who were to be heads of hospitals or training institutions.

### The Rural Midwives' Association and the Midwives Act.

The Rural Midwives' Association, which, it will be remembered, was inaugurated two years ago to help in carrying out the Midwives Act of 1902, by finding and training suitable women for local associations and committees, is engaged, now that the Act has come into operation, in endeavouring to ascertain the nature of the difficulties met with by the various local authorities in administrating its provisions in their various districts. Such difficulties, of course, vary with the locality, and the answers to the schedule of questions, when tabulated, will form the basis of a discussion, during which, it is hoped, that some solutions of the various points raised may be arrived at. The occasion will be the second annual meeting of the Association, to be held probably on May 18th, at the house of Mrs. Gratton, 66 Ennismore Gardens. Already, some 38 County or Borough Councils in England and Wales are engaged, either directly or by delegating their powers in the matter to special committees, in administrating the provisions of the Act, and, provided that the particulars sent in are sufficiently complete and representative, the discussion should prove of no small value in raising points of difficulty, even if it does not result in an immediately satisfactory solution. The Association's midwives are in great demand, many being engaged in advance to work in specified districts; and since the longer period of training required by the Midwives' Board has resulted in the fees at the various recognised training schools being raised, the Association finds its resources heavily taxed. The coming into operation of the Act, with the consequent penalising of any woman unlawfully using the title of midwife, will probably add to the administrative labours of any organisation undertaking the supply of certified midwives, and the present is therefore an opportune time to remind all who are interested in the welfare of the poor in rural districts that the work of the Association is supported by subscriptions, and that it trains working women and others, at a reduced fee to subscribers, to work under associations and committees in all parts of England and Wales. It likewise trains nurses already employed by them in midwifery.

### Appointments.

[No charge is made for announcements under this head, and we are always glad to receive, and publish, appointments. The information, to insure accuracy, should be sent from the nurses themselves, and we cannot undertake to correct official announcements which may happen to be inaccurate. It is essential that in all cases the school of training should be given.]

Birmingham Hospitals' Convalescent Home.—Miss Maud Taylor has been appointed matron. She was trained at the North-Eastern Hospital for Children, Hackney Road, has been sister at the Royal Infirmary, Wigan, sister at West Ham Accident Hospital, and sister at Shoreditch Infirmary.

Chesterfield Infirmary.—Miss Amy Longworth has been appointed night sister. She was trained at Brownlow Hill Infirmary, Liverpool. She has since done private nursing and has been temporary sister at Birkenhead Infirmary. She holds the certificate of the Central Midwives Board.

Eye and Ear Hospital, Tunbridge Wells.—Miss Kathleen M. Nicolas has been appointed matron. She was trained at the Croydon Infirmary and has since been charge nurse at the Branch Seaman's Hospital, Albert Docks, London, E., and sister at the General Hospital, Tunbridge Wells.

Hastings Union Infirmary.—Miss Margaret Lonergan has been appointed assistant-nurse on probation. She was trained at the Nottingham General Hospital by the Meath Workhouse Nursing Association, and has been nurse at Plymouth Union Infirmary.

HOLBORN UNION INFIRMARY.—Miss Elizabeth Wakeham has been appointed assistant-nurse. She was trained for one year at the Mildmay Memorial Hospital as a member of the Meath Workhouse Nursing Association.

ISOLATION HOSPITAL, BROMSGROVE.—Miss M. A. Breed has been appointed sister, and Miss E. T. Davison staff nurse. Miss Breed was trained at Leeds Infirmary and the Rotunda Hospital, Dublin. She has since been sister at Bethnal Green Infirmary, London. Miss Davison was trained at the Borough Hospital, Plymouth, and the South Devon and East Cornwall Hospital, Plymouth.

KASR-EL-AINY HOSPITAL, CAIRO.—Miss Agnes Sowry has been appointed sister. She was trained at St. Thomas's Hospital, London, and has since been sister at Monsall Fever Hospital, Manchester, and the Royal Infirmary, Bristol.

NORTH STAFFORDSHIRE SMALL-POX HOSPITAL.—Mrs. Olivia A. Nixon has been appointed matron. She was trained at the Spittals Workhouse, Newcastle, Staffordshire, and was afterwards charge nurse.

NORWICH ISOLATION HOSPITAL.—Miss Dorothea Webb has been appointed charge-nurse. She was trained at Kidderminster Children's Hospital, and has since been assistant-nurse at the Western Fever Hospital, Fulham, and assistant-nurse at Caistor Union Infirmary, Lincolnshire.

PASSMORE EDWARDS HOSPITAL, WILLESDEN.—Mrs. Carew Hodge has been appointed matron. She was trained at the West Kent Hospital, Maidstone, and Queen Charlotte's Hospital, London. She has since been sister at the Royal Sea-Bathing Hospital, Margate; sister in charge of the out-patient department, Hospital for Women, Soho Square, London; sister and housekeeper at Guy's Hospital; night superintendent at Brompton Hospital for Consumption; assistant-matron at Bethel Hospital, Norwich; and matron of the Eye and Ear Hospital, Tunbridge Wells.

PENRITH COTTAGE HOSPITAL.—Miss Mary Bewsher has been appointed nurse-matron. She was trained at Cumberland Infirmary, where she was afterwards sister, and has since been attached to the Bristol Nurses' Institute.

ROYAL INFIRMARY, BRADFORD.—Miss Maud H. Pidgeon has been appointed housekeeper. She was trained at the Royal United Hospital, Bath, where she was afterwards sister. She has since been charge nurse at the Grove Fever Hospital, Tooting, London, and sister at the Royal Infirmary, Bradford. She has also done private nursing.

ROYAL INFIRMARY, BRADFORD.—Miss Eva E. Bell has been appointed night-superintendent. She was trained at the Royal United Hospital, Bath, and has since been charge nurse at the Grove Fever Hospital, Tooting, London, and sister at Bradford Royal Infirmary.

SHEFFIELD CITY HOSPITAL FOR INFECTIOUS DISEASES.—Miss Flora Morrison has been appointed night superintendent at Lodgemoor Hospital. She was trained at the City Hospital, Aberdeen, and has been theatre sister and temporary night sister at Paisley Infirmary, and had charge of various wards at Ruchill Fever Hospital, Glasgow.

VICTORIA COTTAGE HOSPITAL, ABERGAVENNY.—Miss Edith Burgess has been appointed staff-nurse. She was trained at Lyndhurst Infirmary and the Hospital, Shaftesbury.

### Presentations.

GLOUCESTER DISTRICT NURSING SOCIETY.—Miss Palk, who has lately resigned her post of head maternity nurse at the District Nursing Society, Gloucester, was the recipient on Saturday last of a testimonial which was subscribed to entirely by the patients she has nursed. It consisted of a brass travelling clock, on which is engraved, "To Miss Palk, from her grateful patients, April 1905," a gold curb bracelet, and gold brooch. The presentation was made by three of the patients, who were the collectors.

### Everybody's Opinion.

[Correspondence on all subjects is invited, but we cannot in any way be responsible for the opinions expressed by our correspondents. No communication can be entertained if the name and address of the correspondent are not given as a guarantee of good faith, but not necessarily for publication All correspondents should write on one side of the paper only.

### THE LATE MATRON OF ADDENBROOKE'S HOSPITAL.

MR. RICHARD L. COLES, secretary-superintendent of Addenbrooke's Hospital, Cambridge, writes: Sir,—I am desired by the general committee of the hospital to send you the following copy of the resolution passed by the committee at their weekly meeting: "That the General Committee of Addenbrooke's Hospital lament most deeply the loss which the hospital and the nursing profession have sustained by the death of Miss M. Adams under such specially sad circumstances, and desire to offer to her parents and members of her family their sincere condolence in the terrible calamity which has fallen upon them, assuring them that Miss Adams had not only during the short time she was at the hospital won in a remarkable degree the esteem and confidence of the staff in each department, but had also completely fulfilled, so far as time allowed, the high expectations formed of her from the brilliant testimonials by which her candidature was supported." My committee have the satisfaction of knowing that Miss Adams had testified to the agreeable character of her relations with the staff, and, indeed, with the committee itself, both in letters to friends and in expressions to individuals.

### A NOVEL PROPOSAL.

NURSE E. WOODBURN-HERON writes:—May I reply to "Trained Nurse"? For some years now there has been continual bickering and fault-finding with the untrained or partially trained nurse and her uniform. I, who am only a certificated maternity nurse, have been hoping for some time that the trained nurse would mend her ways before picking holes in her neighbours'. I shall try to show you the appearance the trained nurse presents to the outsider; but first I must mention that I actually know of nurses from three large London hospitals who appear when off duty with aprons under their cloaks and dresses sweeping the streets, both points being against the first and most simple rules of nursing. I presume that the majority of nurses I see in the shops are trained; yet their appalling appearance—bonnet unbrushed; hair, alas! ill-kept, profusely curled or out of curl, and often in need of soap and water; strings soiled, and with a much larger bow than necessary; collar, usually fastened with a brooch of sham stones; cloak much too long, frequently lined with coloured silk, and seldom to match bonnet; dress below cloak, often sweeping the roads and sometimes ornamented with the dirty corners of an apron, while the whole is completed with open-work stockings and high-heeled shoes. Surely the "trained nurse" could make her own appearance a little more professional before continually questioning the right to uniform of other nurses. Of the various London hospitals, there is only one of which I know whose nurses will bear inspection. The Bartholomew nurse is absolutely professional, indeed her immaculate appearance with her neat little bonnet and spotless linen resuscitates in us the dying spark of admiration for the nursing profession. I chose my uniform some years ago, never dreaming of imitating any other nurse. I always wear it, and shall continue to do so, in spite of the sneers and protests of my more accomplished sisters.

### THE TRAINING OF MENTAL NURSES.

"Observer" writes:—Your journal has shown considerable interest in the work of mental nurses of both sexes during the past year, for you have published a series of lectures upon the care of the insane and have also discussed in your columns the various methods and systems of training advocated in the different mental hospitals and asylums, also their bearing upon the position and the *status* of those who devote themselves to this branch of the nursing profession. The present time witnesses an agitation for the special recognition by law of the nursing profession, and a Bill has already this Session been read once in the House of Commons to enable nurses to be registered. The Asylum Workers' Association, a body composed of about 3,000 to 4,000 attendants and nurses engaged at the various asylums in the care of the insane, has obtained recognition upon the Central Board which is to govern this new registration scheme, and the Medico-Psychological Association, which issues a certificate for proficiency in mental nursing, has recently agreed to lengthen the period of training in asylums from two to three years before a candidate can qualify for the examination; also, more recently still, it has agreed that this period of three years must be passed in not more than two asylums. It may not be known to all candidates who seek training in nursing the insane that such training is not general, and that, like hospitals, asylums also vary, some preparing their probationers and nurses for their own certificates, as do such asylums as that at Cheddleton, in Staffordshire, and Berry Wood, in Northampton; others, such as the York Retreat, give both their own medal, after instruction, training, and examination, and also prepare candidates for the nursing certificate of the Medico-Psychological Association. It would be well if all candidates for posts in asylums were to inquire before joining any institution whether there is in that place a recognised system of training and whether facilities are offered for obtaining certificates of fitness to nurse cases of insanity. The examinations of the Medico-Psychological Association take place twice a year, in May and November, a fee of 5s. is charged, and on and after May 1906 the period of training must be three years before candidates can apply to sit for the examination or are qualified to receive the certificate. I have often felt that the great public asylums throughout the country, supported as they are by the rates, should do something more than is at present being done, by supplying private nurses direct to the ratepayers in cases of insanity—of course, for definite payment—such arrangements would also benefit the nurse and would raise the *status* of nursing the insane throughout the country. It would further discourage some of the unworthy private ventures that seek benefit to themselves at the expense of both the nurse and her patient. A nursing institute connected with our public asylums would be of the greatest possible benefit to the public, and could be easily worked in these places without extra cost, for the administrative and clerical staff is already there, and the profits from this scheme would help to provide a much appreciated "gratuity" for nurses who leave after several years' service either to be married, or, as too often happens, in reduced health and without pensions. I think that very much more can be done, for the benefit of the public and to the advantage of the individual nurse, without in any way reducing the efficiency of our public asylums if a scheme were established in these places for supplying private nurses of both sexes. As to the work for the examinations, this consists in elementary anatomy, physiology, hygiene, psychology, first aid, practical work in the sick wards, at the bedside, and preparing reports. The secretary of the Medico-Psychological Association is Dr. Jones, Claybury Asylum, Woodford, Essex, and the registrar is Dr. Miller, County Asylum, Hatton, near Warwick—either of whom would, I am sure, be pleased to answer any inquiries as to training for mental nursing.

### Novelties for Nurses.

(By Our Shopping Correspondent.)

### MESSRS. EGERTON BURNETT'S MATERIALS.

Regularly as spring comes round there arrives from Messrs. Egerton Burnett a large and varied assortment of materials, and this year I notice an innovation of special interest to my readers. This is the establishment of a department for making up nurses' dresses from measurements sent by post, either in washing materials or woollen fabrics. The bodices of the former are lined throughout with good quality washing material, the skirts being unlined, and the cost is from 15s. 11d. for a dress costing 7d. or 7¾d. per yard to 23s. 6d. if the material be more expensive, *i.e.* 1s. 5d. or 1s. 5¾d. The woollen fabric dresses are lined throughout, and cost from 26s. 3d. (material 1s. 2d. to 1s. 3½d. per yard) up to £3 for a dress at the rate of 6s. 9d. or 6s. 11d. per yard. Variation in width, of course, affects the price also, and I should suggest that in writing for patterns this special price list be asked for. The measurements should be taken on the form supplied, or a well-fitting bodice will do, with, of course, skirt measurements. Nurses' cloaks, in all the different shapes, have long been a speciality of this firm, and there is always a large and varied selection of waterproof cloakings, etc., in stock. Special quotations for materials suitable for nurses' use are given to homes, hospitals, and nursing institutions for quantities of 50 yards and upwards. I must also mention the plain tailor-made cycling costumes, coats and skirts, etc., which are made from 16s. 3d. upwards for a coat, to 71s. 9d. for a costume in material costing 6s. 9d. per yard. Measures are registered for use with future orders. With regard to aprons, there is a pure linen for this purpose, 45 inches wide, from 1s. 8d., a strong white pattern at 11d. (40 inches), and a pure brown holland from 8½d. to 1s. 2d. among others; muslin for caps and strings costs from 1s. 1½d., while collars and cuffs are also supplied from 5¾d. and 6¾d. respectively. Nurses' aprons, by the way, can be had ready made with square bibs for 2s. 6d. and 2s. 11½d. Among other novelties I notice a "picnic rug" which would be a very useful present for a nurse going to private cases, spreading on the sands, etc.; it is made in eight different shades, and weighs under 2 lbs. The price is 3s. 11d. But nurses are not always on duty, and they will be glad to know that the patterns of materials for "off-duty" dresses and blouses are as dainty as ever. Mention must be made of the printed voiles for summer costumes, in checks, spots, and floral designs, among which the "Cyclamen" is especially pretty, and there is a beautiful silk-weft zephyr at 1s. 6½d., and a spotted crêpe at 3s. 6½d. among a variety of other materials. For costumes, the "Carlisle," in mouse-brown, is very tempting, as are the "Cromarty," Matlock," and "Montrose," and the "Badminton," "Lonsdale," etc., for smart dresses, and the strong serviceable linens for summer; while for pretty blouses there is such a varied selection of muslins, delaines, etc., that I can only advise my readers to send for patterns to Messrs. Egerton Burnett, Wellington, Somerset, who will send them post free to any address.

### BONNETS, CLOAKS, AND APRONS.

Mr. Joseph Moore, of the Belfast Linen Warehouse, Leeds and Bradford, sends me some specimens of nurses' bonnets, cloaks, and aprons. To take the cloaks first. These are in two styles—one, the Queen, is a circular shape, the other being made with a separate cape. Both are in summer serge of a peculiarly light weight. The price of the first is 14s. 11d. in dark blue, and that of the second 21s., in black, and 26s. 6d. in blue. They are well made, and should commend themselves to nurses seeking a nicely-fitting cloak at a moderate price. The specimens of aprons include a strong linen overall described as an "operating overall," the meaning, of course, being that it is intended to be worn by the theatre nurse. This is a most practical garment, completely covering the dress, and with elbow sleeves, thus allowing for the arms to be left bare to the elbow. It is made in a yoke, fastens with two buttons at the back, and a band meeting in front. The price is 7s. 9d. It is well and strongly made of unbleached linen. Another apron is of strong union, for ordinary ward use. It has a shaped bib ending in shoulder straps, ample in width, and costing 2s. 6d.; while for 3s. 9d. an apron of hem-stitched linen, with a square bib, is certainly well worth the price. There are others at 2s. 3d. and 2s. 6d., of very good value. All the aprons sent are supplied with sensible pockets, a most necessary feature, as every nurse knows. The bonnets are in four styles, only one being adorned with the unnecessary and inconvenient (even if harmless) streamer in black gauze. This is the most expensive of the four, and, frankly speaking, the least pleasing, although, of course, the regulations by which a nurse is bound may occasionally require her to wear a head-gear of a more ornamental type than the plain and useful "Dora," "Princess," or "Albion," all of which commend themselves by their simplicity. The first is in blue straw, and has a broad band of blue velvet round the front, something after the style of the "Dora" cap, while a folded piece of the same completes the trimming. The "Dora" is 10s. 6d. The "Princess," another pretty blue straw bonnet, has a neat bow of blue velvet, and a ruche of the same as an edging; while the "Albion," 10s. 6d., is a slightly different shape in brown straw, trimmed with velvet to match. Each of those mentioned is complete with the exception of strings, and the first-named has an edging of white frilling inside the brim. Mention must also be made of the caps, the prettiest, and also the plainest, being the "Dora," simplicity itself, to wash and iron; while the "Queen," with an edging of embroidery, also opens out quite flat, and runs the "Dora" very closely. Other shapes in muslin and lawn are made up to meet the requirements of the various hospitals, and all are very moderate in price. Nurses should send for price lists, not only of the articles mentioned, but of the many little accessories, such as belts, strings, collars and cuffs, etc., which this firm supplies ready made. The address is 36-40 Albion Street, Leeds, and North Parade, Bradford.

### "NON-FLAM."

The dangers of the ordinary flannelette are being constantly brought to notice through the columns of the daily press, while even in the "Nursing Section" the deaths of nurses through fire, brought about by means of this highly inflammable material, have more than once been recorded. The invention, therefore, of a flannelette which is at once soft, warm, durable, and inexpensive, but without the dangerous property to which the coroners so frequently call attention, is a real boon. "Non-flam," on a practical test, does not "catch fire" in the way that the ordinary flannelette does; one has to hold the match to it for some time to make it burn, and even then it only smoulders, instead of bursting into a flame, as does ordinary flannelette. The value, therefore, of this invention is very great, and nurses would do well not only to recommend it for children with whom they may have to do as patients, but to make use of it themselves in place of the ordinary material. The inventors claim for it that the same process which makes it non-inflammable also renders it aseptic and sterile, so that germs cannot exist upon it. This, of course, is another matter, and requires a much more elaborate test, but by sending for a free sample nurses can at any rate prove for themselves that the risk of its catching fire is exceedingly small. The address of the patentees is "Non-Flam," Desk 50, Aytoun Street, Manchester, and they will no doubt also state the price, which is very little more than that of the ordinary flannelette.

### WASHING DRESS MATERIALS.

Some excellent materials for nurses' washing dresses are being shown by Messrs. Christopher Williamson, among which I should like to call attention to the "superior washing Oxfords." These are specially suitable for uniform wear, and are both strong and cheap without being crude in colour. They are 29 inches wide, and the price is only 6¾d. per yard. The Veronica dress Oxford is the same price, but 2 inches narrower, and is made in fancy and pink stripes, as well as in check, the one first mentioned being in plain colours only. Then there is a very strong Wynburg regatta, a speciality fast dye, which this firm recommends as the best washing fabric for nurses' uniform dresses, and certainly the piece before me is equal in every way to the description given of it. It is 30 inches wide, of a peculiarly pretty shade of blue, and costs 9¾d. a yard. Other materials are lustre gingham, 7¾d. per yard; Haslar regatta, a very serviceable washing fabric, at the same price; and a hair-cord gingham, double width, at 10¾d., a most useful-looking material. A cheap but pretty summer dress could be made of the "new flaked union linen," at 4¾d.; it can be had in various colours, including grey and cream; or there is a costume linen at 6¾d., of a coarser texture, shrunk, and made in choice shades. The coloured linens, about a yard wide, at 1s. 1½d., are particularly pretty for private dress. The same firm supplies also a variety of drills, skirtings, flannels, etc., as well as sheetings, towellings, and other house requisites. The address is 91 Edgware Road, London, W., and patterns will be sent, post free, on application.

### TRAVEL NOTES AND QUERIES.

By our Travel Correspondent.

Switzerland in September (Margaretta).—I am afraid the money at your disposal is not sufficient for the trip. Lucerne or Interlaken would be the cheapest place to go to, and the journey, second class return to Lucerne, is £5 2s. 4d. from London. You might get accommodation at 5s. per day, but, you see, counting tips on the journeys and at hotels, the money will not last out. I think it somewhat a mistake (even if money is no object) to go to Switzerland so late in the year. By six o'clock at the latest you must be indoors, because you can see nothing outside. Then it is an immense journey to take for only 13 or 14 days. Why not go to Belgium and see some of the towns there? it is a short and inexpensive journey; or to the Bay of St. Malo in Brittany? Let me hear what you decide and I will help you to the cheapest arrangements.

Bruges in July (Old Maid).—You have decided wisely. Let me hear again and tell me how much you can each afford to spend and what your tastes are, and I will tell you what excursions to make and what to avoid. You would, I think, do wisely to divide your holiday between Bruges and Brussels, from which two centres you will be able to visit all the chief points of interest. When I know your monetary resources, how long your holiday is to last, and something of your tastes, I can give you more useful advice.

Holiday in Jersey (District Nurse).—The climate is very little different from that of the South of England, it is mild but not very warm, and the sun shines more. There is plenty of good bathing at St. Brelade's Bay and in St. Aubin's Bay. There is a very comfortable inn at St. Brelade, charging 6s. 6d. per day; and at St. Aubin the Terminus Hotel charges 6s. There are numerous lodgings at reasonable prices. It would be well to go to one of these hotels for a night and look round for a lodging.

Rules in Regard to Correspondence for this Section.—All questioners must use a pseudonym for publication, but the communication must also bear the writer's own name and address as well, which will be regarded as confidential. All such communications to be addressed "Travel Correspondent, 28 Southampton Street, Strand." No charge will be made for inserting and answering questions in the inquiry column, and all will be answered in rotation as space permits. If an answer by letter is required, a stamped and addressed envelope must be enclosed, together with 2s. 6d., which fee will be devoted to the objects of "The Hospital" Convalescent Fund. Ten days must be allowed before an answer can be published.

### Notes and Queries.

**REGULATIONS.**

**The Editor is always willing to answer in this column, without any fee, all reasonable questions, as soon as possible.**

**But the following rules must be carefully observed.**

1. **Every communication must be accompanied by the name and address of the writer.**
2. **The question must always bear upon nursing, directly or indirectly.**

**If an answer is required by letter a fee of half-a-crown must be enclosed with the note containing the inquiry.**

*Starch Enema and Abdominal Sponges.*

(20) 1. Will you kindly tell me if a starch enema should be made with boiling water and then allowed to become tepid before injection, or should it be made with cold water and stood in warm water until it becomes tepid? 2. Will you also tell me how to prepare new abdominal sponges?—*Sunlight.*

1. A starch enema should be made with boiling water and allowed to cool to the desired temperature. 2. If the sponges are new they must be thoroughly shaken and beaten to get rid of the sand. To remove the pieces of coral and shell they should be soaked for 24 hours in a solution of hydrochloric acid and water. This is made by adding a drachm of strong acid to a pint of water. The mixing must be done with extreme care and by an experienced person, or the acid may splash into the eye. They are then washed and squeezed out in warm water (temperature 100° F.) which has been boiled and left to cool in a covered vessel to ensure its sterility. From this they are transferred for half an hour to a warm solution of ordinary washing soda and water, for the removal of any fat or albumen. The soda solution may have to be repeated several times before it is removed by again rinsing in warm sterilised water (temperature 100°F.) and the sponges are immersed in cold solution of sulphurous acid (1 in 5) for 12 hours for a final bleaching and sterilisation. During this stage a plate is placed over the sponges to sink them in the solution, otherwise they are apt to become discoloured. Lastly they are washed out with sterilised water, squeezed as dry as possible, and placed in carbolic lotion (1 in 20) ready for the operation.

*Gossamer.*

(21) In a recent number of The Hospital you spoke of a new washing material for nurses' veils (Gossamer), and highly recommended it. I cannot find the paragraph, and should be greatly obliged by your kindly telling me the name of the firm who manufacture it?—*A. J. E.*

Messrs. E. and R. Garrould, Edgware Road, W.

*Feeble-minded Lady.*

(22) Can you give me addresses of homes suitable for a feeble-minded lady who is between 30 and 40 years of age.—*L. Y.*

The National Association for Promoting the Welfare of the Feeble-minded, 53 Victoria Street, S.W., may be able to advise you.

*Uniform.*

(23) Will you kindly tell me if holding a certificate for massage and electricity I may wear a nurse's uniform?—*Anxious.*

Anyone may wear a nurse's uniform.

*Convalescent Home.*

(24) In a recent Hospital you spoke of the need of a home for patients just out of hospital. I think of starting one. Can you advise me?—*F. M. K.*

We fear that your proposed home would not meet the necessity. There are plenty of homes now existing for those who can pay well.

*Trained Nurses on Board Ship.*

(25) Will you kindly tell me if any line of ships take trained nurses on board to attend passengers, and to whom to apply?—*W. B.*

Will you kindly tell me which line of steamers carries trained nurses, and how I can obtain an appointment of this kind?—*M. L.*

There is a movement on foot having this object in view. You might write to Miss Penn, the Cottage, New Shoreham, Sussex.

**Handbooks for Nurses.**

| | Post Free. | |
|---|---|---|
| "How to Become a Nurse: How and Where to Train." | 2s. | 4d. |
| "The Nurses' Dictionary" (Pronouncing) ... ... | 2s. | 0d. |
| "Nursing: its Theory and Practice" (Lewis.) ... ... | 3s. | 6d. |
| "The Light Treatment" (just published) ... ... | 2s. | 6d. |
| "A Complete Handbook of Midwifery." (Watson.) ... | 6s. | 4d. |

Of all booksellers or of the Scientific Press, Limited, 28 & 29 Southampton Street, Strand, London, W.C.

### For Reading to the Sick.

### HOLY WEEK.

The stars grow brighter as the night darkens. As the lights of earth are put out one by one, the countenance of heaven makes plainer revelations. Grace makes a very sunset of what to nature is the most impenetrable darkness, and the plaintive strains of the *Miserere* merge, in spite of humility, into songs of triumph; for the walls between the dying soul and the heavenly Jerusalem are so nearly fretted through, that the loud Alleluias mingle with the contrite love whose eyes are closing on the Cross.—*F. N. Faber.*

Our pains are portioned to our powers—
His hand may hurt, but cannot harm—
But, if the Cross be on us laid, and our soul's Crown of Thorns be made,
Then, sure, 'twere best to bear the Cross, nor lightly fling the thorns behind,
Lest we grow happy—by the loss of what was noblest in the mind!
Here—in the ruins of my years—Master, I thank Thee through my tears.
Thou suffered'st here, and didst not fail—Thy bleeding feet these paths have trod—
But Thou wert strong, and I am frail; and I am man, and Thou art God!
How I have striven, Thou know'st! Forgive how I have failed, Who saw'st me strive!—*Lytton.*

Among all the deadly sorrows of His most bitter Passion, this, even this, seemeth to be the greatest of all, and that which did most affect Him, even the grief of the slender reckoning most men have it in, as if He had done or suffered nothing at all for them. For lo! of all the sharp pains He endureth He complaineth not, but of this he complaineth; of no regard; that which grieveth Him most, that which most He moaneth is this as if He said: "All that I suffer, I suffer willingly, if this I may find at your hands, regard." And, indeed, the pain of the body is but the body of pain; the very soul of sorrow and pain is the soul's sorrow and pain. By Thine unknown sorrows and sufferings, have mercy on us and save us.—*Bishop Andrews.*

Give me the lowest place; not that I dare
Ask for that lowest place, but Thou has died
That I might live and share
Thy glory by Thy side.

Give me the lowest place: or if for me
That lowest place too high, make one more low
Where I may sit and see
My God, and love Thee so.—*Christina Rossetti.*

The tendency of man's fancy to connect magnitude of space and time with the real intrinsic magnitude of events is but a delusion. Three hours are but a drop in the ocean of eternity, and a wooden cross but a point in the infinity of space, and yet they were sufficient to complete the great miracle of man's redemption.—*W. Sewell.*

There is no grief that ever wasted man,
But finds its Hour here in thine awful Week.—*Keble.*

## Figures and Tables

**Figure f1:**
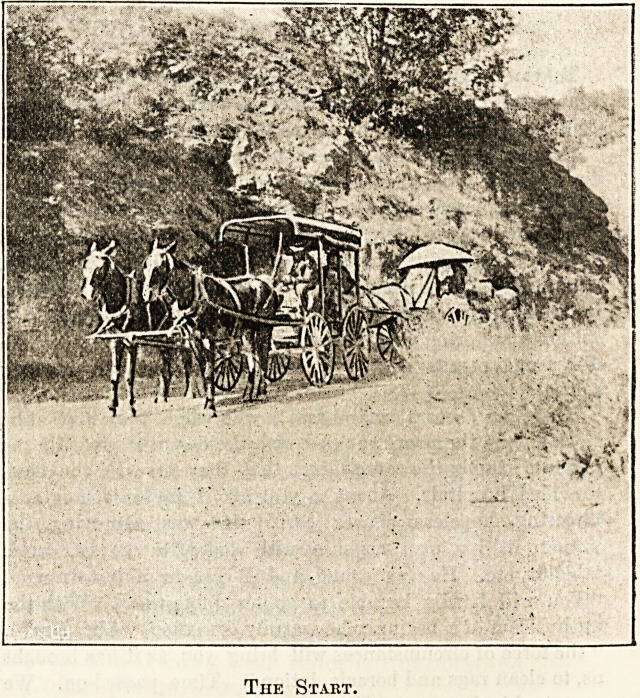


**Figure f2:**
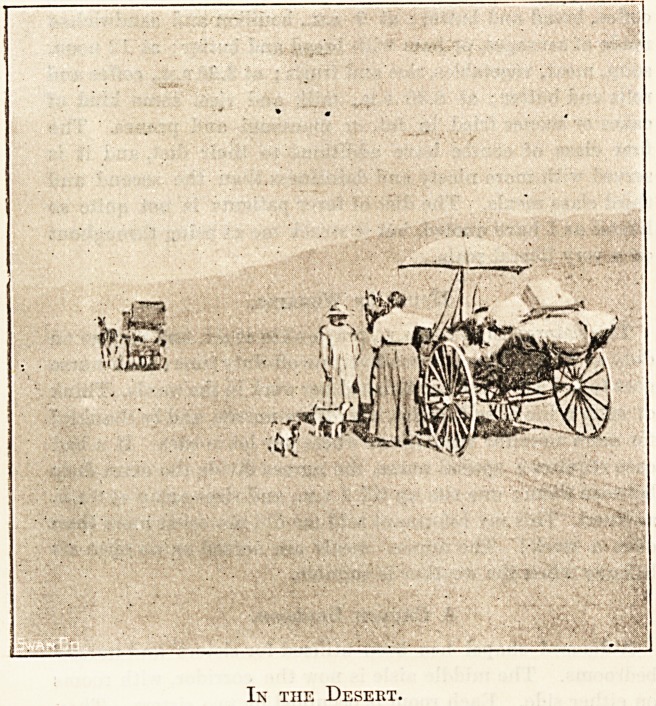


**Figure f3:**
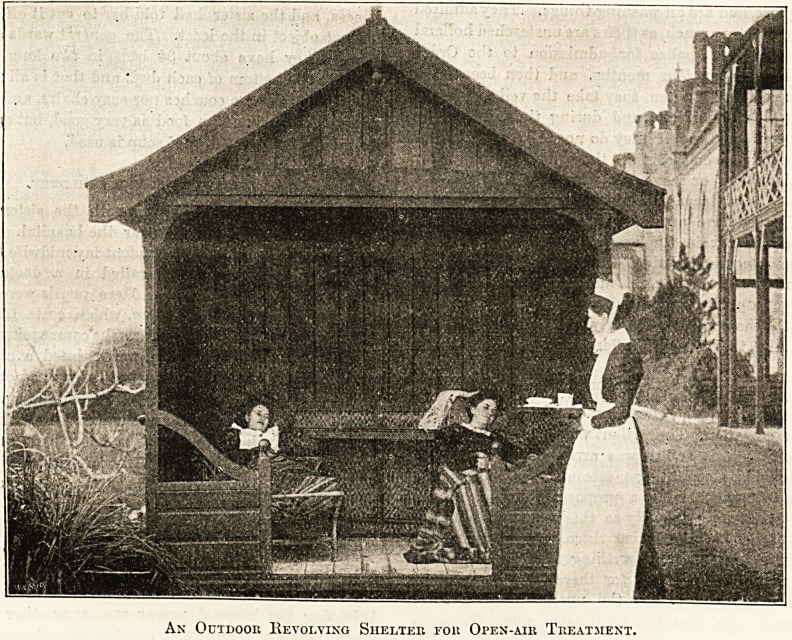


**Figure f4:**